# Ferromagnetic–Antiferromagnetic Coupling in Gas‐Phase Synthesized M(Fe, Co, and Ni)–Cr Nanoparticles for Next‐Generation Magnetic Applications

**DOI:** 10.1002/advs.202403708

**Published:** 2024-09-24

**Authors:** Murtaza Bohra, Stefanos Giaremis, Abisegapriyan KS, Stella Mathioudaki, Joseph Kioseoglou, Panagiotis Grammatikopoulos

**Affiliations:** ^1^ Physics Department, School of Engineering Mahindra University Survey Number 62/1A, Bahadurpally Jeedimetla Hyderabad Telangana 500043 India; ^2^ School of Physics Department of Condensed Matter and Materials Physics Aristotle University of Thessaloniki Thessaloniki 54124 Greece; ^3^ Center for Interdisciplinary Research & Innovation Aristotle University of Thessaloniki Thessaloniki 54124 Greece; ^4^ Materials Science and Engineering Guangdong Technion – Israel Institute of Technology Shantou Guangdong 515063 China; ^5^ Acumen Public Affairs Rue de la Loi 227 Brussels 1000 Belgium; ^6^ Guangdong Provincial Key Laboratory of Materials and Technologies for Energy Conversion Guangdong Technion – Israel Institute of Technology Shantou Guangdong 515063 China

**Keywords:** Curie temperature, exchange bias, ferromagnetic–antiferromagnetic coupling, magnetic nanoparticles, magnetocaloric effect

## Abstract

Combining ferromagnetic–antiferromagnetic materials in nanoalloys (i.e., nanoparticles, NPs, containing more than one element) can create a diverse landscape of potential electronic structures. As a result, a number of their magnetic properties can be manipulated, such as the exchange bias between NP core and shell, the Curie temperature of nanoparticulated samples, or their magnetocaloric effect. In this work, such a family of materials (namely *M*–Cr NPs where *M* is Fe, Co, Ni, or some combination of them) is reviewed with respect to the tunability of their magnetic properties via optimized doping with Cr up to its solubility limit. To this end, gas‐phase synthesis has proven a most effective method, allowing excellent control over the physical structure, composition, and chemical ordering of fabricated NPs by appropriately selecting various deposition parameters. Recent advances in this field (both experimental and computational) are distilled to provide a better understanding of the underlying physical laws and point toward new directions for cutting‐edge technological applications. For each property, a relevant potential application is associated, such as memory cells and recording heads, induced hyperthermia treatment, and magnetic cooling, respectively, aspiring to help connect the output of fundamental and applied research with current real‐world challenges.

## Graphical abstract

Grammatikopoulos and co‐workers highlight fascinating magnetic phenomena that emerge by combining ferromagnetic–antiferromagnetic metals in nanoparticles (case‐study: *M*–Cr). They advocate for gas‐phase synthesis as a means to fine‐tune their configuration and manipulate properties such as the exchange bias, Curie temperature, or magnetocaloric effect, with potential applications in memory cells and recording heads, induced hyperthermia treatment, magnetic cooling, and more.

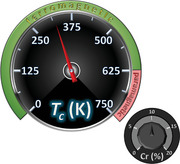

## 1 Introduction

The growth of magnetic materials and their incorporation in functional devices is a central field in modern materials science. Simultaneous advances in fabrication methods and computer modeling techniques triggered the study of a great variety of magnetic materials ranging from binary to multicomponent alloys and from diluted magnetic semiconductors to magnetic oxides.^[^ ^1^ ^]^ Multifunctional materials integrating a diversity of properties (sometimes competing with each other) are of specific interest for numerous applications including spintronics and magneto‐electronics, magnetic hyperthermia, and magnetic cooling because they can enhance the functionality of devices.^[^ ^2^, ^3^, ^4^ ^]^

Magnetic *nano*materials, in particular, are fundamentally interesting because their magnetic behavior is directly related to their size in a way bulk materials are not. The nanoscale is the exciting boundary between the macroscopic and the quantum realms, where nanomaterial properties may be influenced by direct manifestations of quantum mechanics or merely follow (semi)classical physics. A good example where downsizing to the nano‐regime leads to deviations from common properties as we know from everyday experience is the formation of single‐magnetic‐domain NPs below certain size thresholds.^[^ ^5^ ^]^ Such monodomains can be regarded as “superspins,” whose physics are not dissimilar to those governing individual spin interactions in bulk materials (e.g., exchange or dipolar interactions).

The culprits behind such deviations are the quantum‐size effect and the high surface‐to‐volume ratio of NPs.^[^ ^6^ ^]^ Both affect the energy bands (d and f bands for transition and rare earth metals, respectively) of magnetic NPs, which, in turn, modifies the magnetic behavior of the nanoparticulate assemblies.^[^ ^7^ ^]^ Depending on their structural characteristics such as NP sizes or distances, different interactions between “superspins” can generate a whole range of magnetic behaviors, from ferromagnetic (FM) and antiferromagnetic (AFM) to spin‐glassy and paramagnetic (PM).

## 2 A Class of Materials with Tunable Magnetic Properties

Magnetic alloys with mixed FM and AFM exchange interactions are of particular interest. If a FM material is mixed with an AFM material, several distinguishable magnetic configurations may occur, potentially leading to magnetic frustration.^[^ ^8^ ^]^ The latter occurs when it is not possible for some atoms to assume an unambiguous FM or AFM state with reference to all their neighbors of the same or different atom type. If, for example, two AFM Cr atoms are immersed in a FM Fe matrix at distant positions, their moments will be aligned in an anti‐parallel fashion relative to that of the Fe atoms and no frustration will be present. If, however, they are in neighboring sites, they will have to either align antiferromagnetically to the Fe matrix (meaning they sacrifice their AFM alignment with each other), or vice versa.^[^ ^9^ ^]^ Clearly, the probability of this happening is a direct function of the Cr concentration, underlining the sensitivity of the alloy's magnetic properties on its composition. As a result, binary M─Cr alloys (where M is FM Fe, Co, or Ni) form a class of materials with both fundamental science and application interest, widely studied due to the diverse magnetic phenomena they may display.

### 2.1 Effect of Electronic Structure on the Magnetization of Alloys

A way to describe the electronic structure of alloys is by their average number of valence electrons per atom, *n*_v_. Any crystallographic phase can only accommodate a certain concentration of valence electrons. This means that modifications in the concentration of electrons may trigger structural changes in various alloys. Based on the band theory of nearly free electrons, Hume–Rothery rules indicate *n*_v_ values of 1.36, 1.48, and 1.69 for *fcc*, *bcc*, and *hcp* structures, respectively.^[^ ^10^ ^]^ Thus, *bcc* structures become more favorable than *fcc* with increasing *n*_v_ values, and *hcp* structures even more so. An intuitive physical explanation relates the required electron concentration for the appearance of a new phase with that for the Fermi surface to make contact with the boundary of the Brillouin zone. Energy bands split in two near the zone boundary, meaning that if more electrons are added to an alloy, they will occupy either the upper band or high‐energy states of the lower band; thus, increasing the overall energy of the system. Instead, it may be energetically less costly if the crystal changes its structure into one with a Fermi surface of larger volume (i.e., one that can accommodate more electrons).^[^ ^10^ ^]^

In transition metals such as Fe, Co, and Ni, which are of interest here, there is another implication. According to the empirical (and approximate) Madelung rule, their inner 3*d* orbitals are filled after the valence‐shell 4*s* orbitals, even though they have lower energy. Considering that the density of states (DoS) at the top of the *d*‐bands are remarkably high, the *d* orbitals determine the magnetic character of such metals and their alloys. The valence *s* and *p* orbitals contribution is negligible. For example, the net magnetic moment in Ni (electronic structure: 3*d* ^8^ 4*s* ^2^) is determined by the possibility for a 0.54 hole content in the 3*d*‐down band, asymmetrically to the 3*d‐*up band due to an exchange interaction. This accounts for the FM nature of pure Ni below its *T*_C_. Above *T*_C_, the net magnetic moment is zero as the hole content is equally shared by the 3*d*‐up and 3*d*‐down bands.^[^ ^10^ ^]^

**Figure 1:**
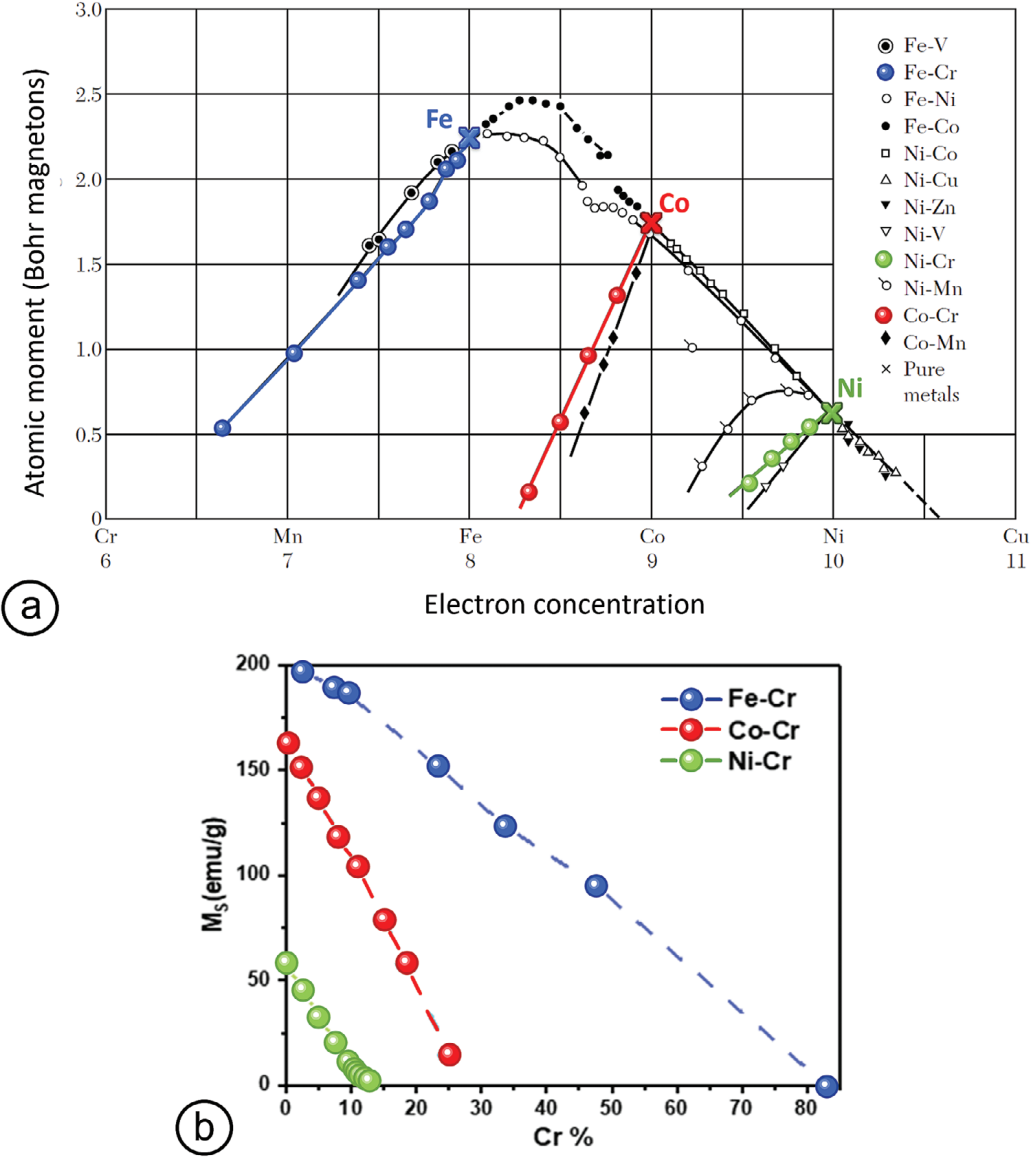
a) Slater–Pauling curve for 3d transition metals and their alloys as a function of the number of their valence‐band electrons. Adapted with permission.^[^ ^10^ ^]^ Copyright 2004, John Wiley and Sons. b) *M*_s_ versus Cr at% for the three *M*–Cr alloys of interest plotted for this review from data points in refs. [14, 15, 16].

When binary alloys are formed, an intuitive (even if not totally accurate; see here for generalizations^[^ ^11^ ^]^) description of their magnetic behavior is the Slater–Pauling (SP) rule, which associates the total‐spin magnetic moment, *M*_t_, to the total number of valence electrons, *n*_v_.^[^ ^12^, ^13^ ^]^ Several binary alloy systems follow the SP rule because their *M*_t_ scales linearly with *n*_v_, as shown in **Figure** **1**a. On the left‐hand‐side in this figure, *M*_t_ increases by 1 µ_B_ with each valence electron added in the compound, occupying spin‐up states only (the majority *d*‐band is filled because of Hund's rule). On the right‐hand‐side, *M*_t_ decreases by 1 µ_B_ each time an extra valence electron occupies exclusively spin‐down states.

For example, when Ni is alloyed with Cr (electronic structure: 3*d* ^5^ 4*s* ^1^), the *n*_v_ value of the alloy (corresponding to the effective magneton number per either type of atom) decreases. With increasing Cr concentration, the hole content asymmetry of Ni gradually closes down, and the alloy becomes less magnetic until it reaches the point where net magnetic moment becomes zero,^[^ ^10^ ^]^ with its saturation magnetization going through zero at ≈15 at% Cr.^[^ ^14^ ^]^ As a result, Ni–Cr (and several other alloy systems such as Co–Cr, as well as Ni–Mn and Ni–Fe) grow distinct branches in the SP plot, which is indicative of AFM ordering.^[^ ^13^ ^]^

The marked departure from the SP plot that can be seen when alloying Cr with itinerant FM metals such as Ni or Co leads to a sharp decrease in both saturation magnetization and magnetic ordering temperature, as shown in Figure 1b.^[^ ^16^ ^]^ A successful explanation for this behavior of 3*d*–3*d* alloy dilution for low impurity concentrations is based on Friedel's Virtual Bound States (VBS) theory.^[^ ^17^ ^]^ The SP rule also directly connects the electronic with the magnetic properties of *M*–Cr alloys; and thus, allows to determine the electro–magnetic character through magnetization measurements. Therefore, doping with Cr provides a considerable tuning capability of the magnetic properties of *M*–Cr solid solutions up to the Cr solubility limit of their respective bulk phase diagram, which avails a variety of technological applications. This is the main theme of this review article, which will recur oftentimes in the following sections.

### 2.2 Bulk Phase Diagrams of *M*–Cr Systems

To guide our study, an overview of the three systems of interest based on their bulk phase diagrams is expedient.

**Figure 2:**
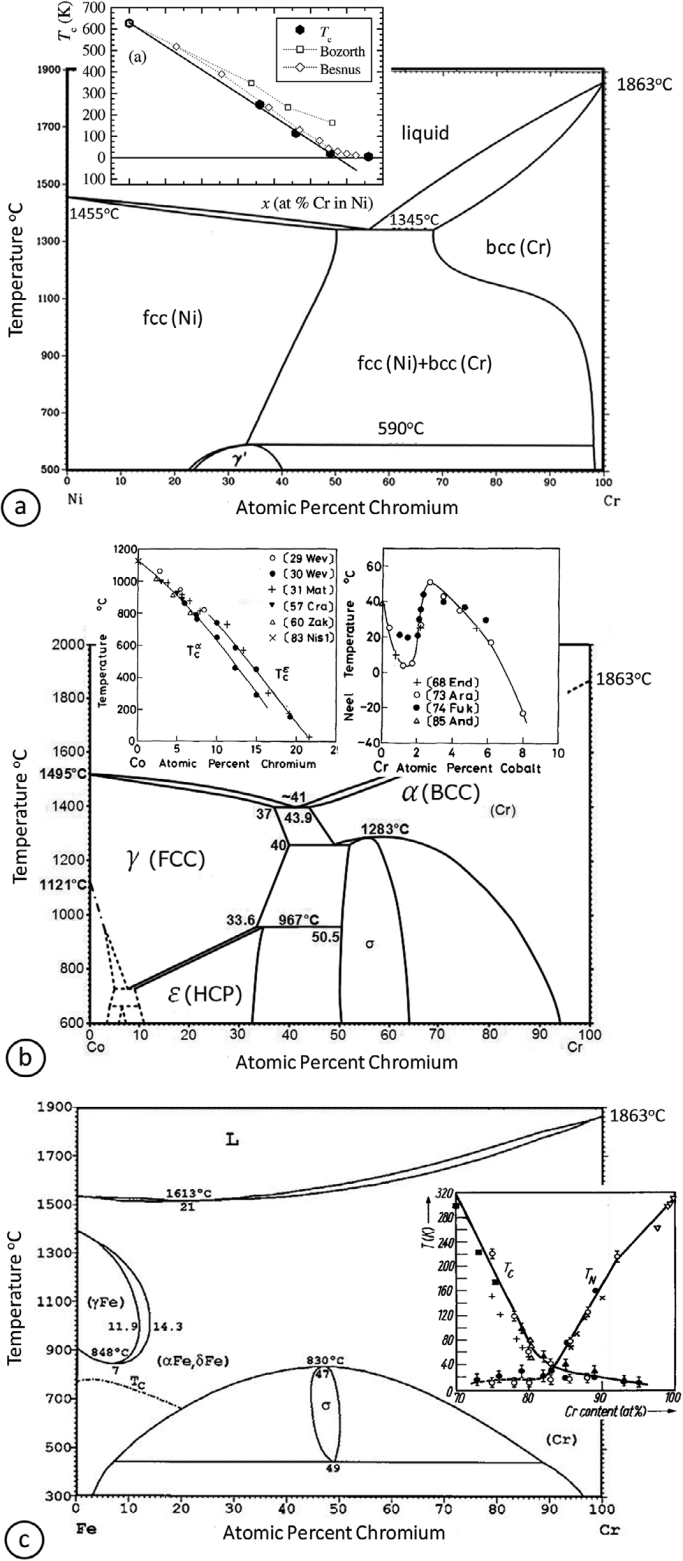
a) Ni–Cr bulk phase diagram. Reproduced with permission.^[^ ^18^ ^]^ Copyright 2010, Springer Nature. The inset shows the *T*_C_ as a function of Cr at% content. Reproduced with permission.^[^ ^19^ ^]^ Copyright 2002, Elsevier. b) Bulk phase diagram of the Co–Cr binary system. The insets show the *T*_C_ dependence of *fcc* (γ‐Co) and *hcp* (ε‐Co) phases on Cr at% and the *T*_N_ dependence on Co at%. Reproduced with permission.^[^ ^20^, ^21^ ^]^ Copyright 1990, Springer Nature. c) Fe–Cr bulk phase diagram. Reproduced with permission.^[^ ^22^ ^]^ Copyright 2019, AIP Publishing. The inset shows the *T*_C_ and *T*_N_ dependence on the Cr at% for the FeCr alloy based on data from various studies. Reproduced with permission.^[^ ^23^ ^]^ Copyright 2006, John Wiley and Sons.

The bulk equilibrium phase diagram of the Ni–Cr system is a typical eutectic phase diagram, showing solid solubility of Cr in Ni up to ≈40 at% Cr (**Figure** **2**a). Below that threshold concentration, Ni–Cr alloys are *fcc* substitutional solid solutions (γ‐phase). At the high‐Cr concentration end of the spectrum, beyond the solubility limit in Ni, the equilibrium structure is a two‐phase mixture of *bcc* Cr/Ni (α‐phase) and *fcc* Ni/Cr (γ‐phase) solid solutions.^[^ ^18^ ^]^ The incorporation of Cr to Ni results in a pronounced magnetization loss and in the aforementioned clear deviation from the main Λ‐shaped SP curve of *M*–Cr alloys. For Ni–Cr alloys, the Curie temperatures (*T*_C_) decrease rapidly, extrapolating to zero at ≈10–15 at% Cr (inset of Figure 2a).^[^ ^16^ ^]^ For *fcc* alloys, where each atom is surrounded by twelve first‐nearest‐neighboring (1NN) atoms, this concentration corresponds to a mean of less than two Cr atoms in the 1NN shell around each Ni atom. Hence, the Cr impurities must affect the Ni matrix profoundly.^[^ ^16^ ^]^

The low critical Cr concentration for the disappearance of ferromagnetism renders this system very attractive for new potential magnetic applications. The high Cr‐concentration range (Cr ≥ 20 at%), where ferromagnetism gradually shifts toward paramagnetism, has also been investigated in depth because of other favorable properties such as corrosion resistance, low oxidation rate, high strength/hardness, and high temperature resistance heating (e.g., nichrome^[^ ^24^ ^]^). However, in the current review, where we focus on the magnetic properties, we consider nanoalloys within the pure‐Ni to 15 at% Cr concentration range.

The bulk phase diagram of the Co–Cr system (shown in Figure 2b) shows four stable phases: namely, *fcc* (*γ*‐Co), *hcp* (*ε*‐Co), *bcc* (α‐Cr), and the σ‐phase.^[^ ^25^, ^26^ ^]^ With the addition of Cr, the saturation magnetization of Co decreases, and the resultant alloy displays a moderately stable *hcp* phase at Cr concentration lower than 40 at%.^[^ ^15^ ^]^ In the composition range 0 < *x*_Cr_ < 0.3 alloys have *fcc* structures that are stable at high temperatures and martensitically transform into *hcp* phases at low temperatures. The *T*_C_ of the alloys of *hcp* structure is higher than that of *fcc* structure for *x*_Cr_ > 0.1 (insets of Figure 2b). For lower Cr concentrations, the *hcp* phase converts into the *fcc* phase before its Curie temperature is reached.

The Fe–Cr system shows noteworthy magnetic behavior, since it is FM in the Fe‐rich end of the composition spectrum and AFM in the Cr‐rich one.^[^ ^22^, ^23^ ^]^ The magnetic coupling depends heavily on both the alloy's composition and its chemical ordering state. The equilibrium phase diagram for the Fe–Cr system (Figure 2c) generally consists of a FM *bcc α*‐FeCr phase, a relatively smaller ferrimagnetic (FiM) tetragonal *σ*‐FeCr phase, and some AFM phases. The dashed line at ≈700 °C (denoted as *T*_C_ in the figure) indicates the transition from the ordered FM α‐phase to a disordered α–δ paramagnetic (PM) phase. The Fe–Cr alloys in the concentration range 80–85 at% Cr undergo a transition from ferromagnetism to anti‐ferromagnetism (see inset of Figure 2c for the Curie and Néel temperature dependencies on Cr content: *T*_C_ and *T*_N_, respectively). In general, the proportion of the σ‐FeCr phase, which consists of equiatomic intermetallic compounds, depends on the Fe‐to‐Cr ratio during the preparation process and is maximized when this ratio reaches an appropriate value.^[^ ^27^ ^]^

However, the picture is considerably more complex for *nano*alloys (i.e., NPs consisting of more than one elements), where numerous nonequilibrium phases have been reported for fine particles. Typically, nanoalloys quenched to room temperature (RT) are in a metastable state and their properties are in many respects determined by the details of their preparation; this is the subject of the next section.

## 3 Gas‐Phase Synthesis of *M*–Cr Nanoalloys

It is almost an introductory section cliché that the properties of materials at the nanoscale can be substantially different from those of their bulk counterparts. Nevertheless, the magnetic properties of alloy NP samples are indeed highly sensitive to the overall sample coverage (determining distances from neighboring particles), as well as to individual particle sizes, composition, and chemical order (involving processes such as elemental surface segregation and oxidation). As a result, *M*–Cr alloy NPs avail unique possible applications compared with *M*–Cr films or bulk alloys, stemming from their diverse phase composition, potentially complex structure, and most interestingly for our current topic, from magnetic exchange interaction between particles.^[^ ^28^ ^]^

To date, NP fabrication usually follows wet chemistry routes. Common magnetic NPs are typically prepared by some sol–gel synthesis process, including thermal decomposition at high temperatures, solvothermal, polyol, and chemical co‐precipitation.^[^ ^29^ ^]^ Such methodologies enable the high‐yield formation of monodisperse NPs of various controllable shapes with high saturation magnetization values.^[^ ^30^, ^31^ ^]^ *M*–Cr nanoalloy fabrication (e.g., NiCr NPs,^[^ ^32^ ^]^ see hyperthermia section, below), in particular, has been reported following the microemulsion route. While the technique is simple, versatile, low‐cost, and succeeds in producing small NPs (in the order of few nm or tens of nm in diameter), it cannot guarantee homogeneity either in terms of size or composition, while post‐fabrication thermal treatment has been necessary to induce crystallization to the NPs. Alternatively, when the requirement for small particle size is not stringent, the top–down mechanical milling approach is commonly used for the fabrication of micro‐ or nano‐powders via friction/impact of the desired material with grinding spheres. Although variants exist (e.g., high energy or planetary ball milling^[^ ^33^ ^]^), they share the same limitations: low work efficiency and high energy consumption, potential contamination during the process, and relatively large particle sizes (of the order of 100 nm to 1 µm in diameter).

Gas‐phase synthesis, on the other hand, is a relatively new solvent‐ and effluent‐free technique to fabricate nanomaterials with bespoke features.^[^ ^34^ ^]^ Due to its young age, the related scientific community is still moderately limited compared with that of wet chemistry. However, the current thrust for ecological design and fabrication, along with the fundamental advantages of gas‐phase synthesis (e.g., sample purity, batch homogeneity, etc.) are actively promoting the incorporation of gas‐phase synthesized NPs in industrial‐level applications.^[^ ^35^, ^36^ ^]^ It should be stressed that the properties of the gas‐phase synthesized nanoparticulate films are not always attainable by wet chemistry growth. Gas‐phase synthesis is often the only method that allows simultaneously controlling parameters such as pure cluster‐support interface, NP soft landing, surface coverage, mixing of various elements (if desired), and C‐MOS compatibility. In the next section, two families of gas‐phase syntheses will be discussed for the formation of *M*–Cr nanoalloys: cluster beam deposition (CBD) and aerosol synthesis.

### 3.1 Gas‐Phase Synthesis Methods

**Figure 3:**
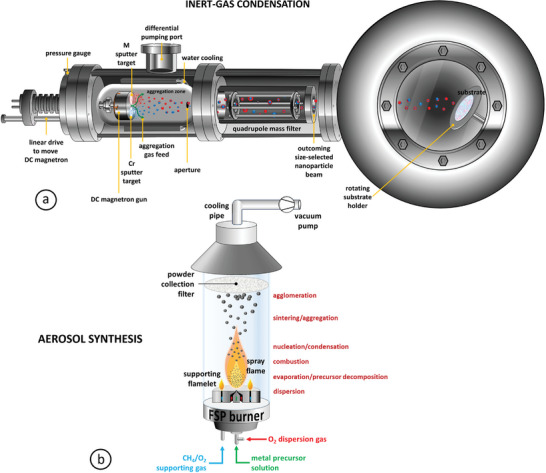
a) Schematic of exemplar magnetron‐sputtering inert‐gas condensation source, a typical CBD‐type source for the fabrication of NPs by design. b) Schematic of exemplar flame spray pyrolysis source, a typical device for the fabrication of aerosols; the various stages of primary particle nucleation and growth, aggregation, and agglomeration are indicated. Numerous variations of both types of NP sources exist.

Cluster beam deposition is a variant of physical vapor deposition (PVD), one of the most widespread bottom–up production methods, which offers a desirable combination of speed, high‐quality, and low‐cost.^[^ ^37^, ^38^, ^39^ ^]^ A common CBD method is magnetron‐sputtering inert‐gas condensation,^[^ ^40^, ^41^, ^42^, ^43^ ^]^ where positively charged ions from a plasma discharge bombard a metallic target, sputtering material away (**Figure** **3**a). For enhanced yield, the electrically conductive plasma is typically adjacent to the target surface by a magnet located just behind it (hence, *magnetron* sputtering). Its attractiveness stems from its innate flexibility: by tuning parameters such as magnetron power, inert‐gas composition and pressure, or NP drift velocity and aggregation zone length, one can control growth rates, NP sizes, or sputtering yield, respectively.^[^ ^44^, ^45^ ^]^ Further, the intricate interplay between the plasma and the number or structure of the NPs enable fine‐tuning of the latter. For example, a secondary plasma exists further downstream from the target,^[^ ^46^, ^47^ ^]^ which determines (and also depends on) the size and density of the produced NPs, as manifested by plasma characteristics such as the electron temperature and spatial density distribution or the plasma and floating potentials.^[^ ^48^, ^49^ ^]^

By using alloy/composite targets or additional sputtering sources, one can design multi‐component NPs with tunable composition and structure (e.g., of mixing or demixing type^[^ ^50^, ^51^ ^]^). In particular, CBD is a very efficient method for the design of core–shell NPs, either by simultaneous co‐sputtering of immiscible materials^[^ ^41^ ^]^ or by in‐flight sequential coating of a second phase on preformed core clusters.^[^ ^52^ ^]^ Alternatively, FM NPs can be embedded in AFM matrices; thus, inducing interfacial magnetic anisotropy.^[^ ^53^, ^54^ ^]^ Introducing oxygen in the aggregation chamber may also result in oxide shell formation; interestingly, either FM or AFM phases may be thus generated, resulting from a sensitive dependence on the degree of oxidation (e.g., CrO_2_ is FM, whereas Cr_2_O_3_ is AFM).^[^ ^55^ ^]^ Further post‐growth or post‐deposition control techniques, such as in situ mass filtration or surface passivation, respectively, also offer a plethora of potential desired configurations.

In aerosol synthesis (e.g., flame spray pyrolysis), appropriate precursors are introduced in a suitable reactor where they turn into gas (Figure 3b). Subsequently, after materials synthesis takes place, the particles are cooled at a selected pace, collected, and if necessary, gas‐treated.^[^ ^56^ ^]^ In the cases discussed below for the study of the exchange bias phenomenon, aerosol particles were produced by gas evaporation of an arc‐melted drop of a specimen containing the initial alloys, deposited and cooled on the walls of a stainless‐steel cylinder, and eventually, passivated with an oxide shell.^[^ ^57^ ^]^

### 3.2 Possibilities for Enhanced Structural Control

Growth of NPs via gas‐phase synthesis methods can lead to structures whose physical and chemical ordering do not have to agree with their equilibrium bulk counterparts, due to kinetic factors often prevailing over energetics. As a result, a multitude of metastable phases can be stabilized at RT. In terms of physical structure, this translates into the possibility to engineer non‐spherical NPs. For example, Zhao et al. reported a kinetics‐based recipe for fabricating *bcc* Fe nanocubes purely by fine‐tuning the thermal environment during growth, without any need to use chemical surfactants to enhance or hinder specific facet growth;^[^ ^58^ ^]^ similar recipes work for other crystallographic groups, too. Anisotropic shapes can be of great value for magnetic applications; for example, by increasing specific absorption rate (SAR) values for hyperthermia applications (see the relevant section below).^[^ ^59^ ^]^

Further, anisotropy can be induced in gas‐phase deposited NPs by their partial coalescence.^[^ ^60^ ^]^ Coalescence can happen in flight;^[^ ^61^, ^62^, ^63^ ^]^ especially for magnetic NPs, M. Zachariah and collaborators recently exploited the magnetization of FM clusters for the vapor‐phase assembly of nanochains with low fractal dimension guided by an external magnetic‐field suitably positioned near the cluster source.^[^ ^64^ ^]^ However, coalescence also likely occurs after deposition (a.k.a. Smoluchowski ripening^[^ ^65^ ^]^) due to the deposition typically occurring in the soft‐landing regime, allowing the particles to roam on the support. This means that the probability of Smoluchowski ripening can, to a great extent, be controlled by surface coverage (via deposition duration).^[^ ^66^ ^]^ Soft landing also allows particles to be easily harvested and dispersed in solutions for further functionalization. In fact, NPs can be directly deposited in liquids,^[^ ^67^ ^]^ doing away with the need for post‐growth harvesting.

The possibility for non‐favorable chemical orderings (i.e., atomic arrangements) is among the main attractions of gas‐phase synthesis. For instance, magnetic NiCr nanoalloys (i.e., 0–15 at% Cr) were grown by magnetron‐sputtering inert‐gas condensation in metastable random solid‐solution configurations. Subsequently, the nanoalloys demonstrated counter‐intuitive post‐growth behavior: full Cr‐surface demixing was observed up to ≈5 at% Cr; whereas, above this concentration, Cr atoms segregated only partially. This configuration would not have been possible had the particles been grown in energetically‐favorable segregated structures in the first place. Most importantly, it had a profound effect on the nanoalloy magnetic properties, as we discuss below (Section 4.2.2), due to the competition between FM and AFM ordering within and among the particles. As a result, tuning the *T*_C_ values of samples within a wide temperature range was possible for applications such as magnetic hyperthermia for cancer treatment.^[^ ^68^ ^]^

## 4 Tunable Magnetic Properties for State‐of‐the‐Art Applications

FM/AFM nanostructures with distinct core and shell magnetic phases demonstrate enhanced (or sometimes novel) magnetic properties and are currently being investigated for potential applications in magnetic random access memories,^[^ ^69^ ^]^ spintronic devices,^[^ ^70^ ^]^ drug delivery,^[^ ^71^ ^]^ tissue engineering,^[^ ^71^ ^]^ cell separation,^[^ ^71^ ^]^ MRI targeted‐cell imaging,^[^ ^72^ ^]^ and hyperthermia cancer treatment.^[^ ^72^ ^]^ In the next sections, we will review some of those and indicate challenges and opportunities regarding the utilization of gas‐phase synthesized *M*–Cr NPs.

### 4.1 Exchange Bias for Spintronics/Magnetic Memory Recording

FM/AFM structures are of particular interest for spintronics devices due to the predicted benefits of non‐volatility, low power consumption, reduced device sizes, and enhanced performance speeds (i.e., for logic operations, data retrieval, etc.).^[^ ^72^, ^73^, ^74^, ^75^ ^]^ In this regard, tuning core/shell nanostructures by regulating their size, shape, surface composition, core and shell thickness, interparticle interactions, and microscopic structure of the interface of any magnetically inhomogeneous system may add substantial value in a number of application‐oriented phenomena.^[^ ^69^, ^70^ ^]^

An important element of FM/AFM candidature for magnetic devices and other applications is the preservation of magnetically ordered states (AFM, FM, FiM) to RT. FM/AFM structures having above‐RT AFM, FM/FiM, superparamagnetic blocking temperature, and magnetoelectric properties are scarcely reported. Conventional FM/AFM structures are characterized by a FM core and an AFM or FiM shell; instead, inverted core–shell NPs comprise AFM cores and FM/FiM shells. Core and shell regions with distinct magnetic phases may interact through the exchange bias effect, a fact particularly attractive for magnetic device and spintronics applications. The inverted FM/AFM structures are especially promising because their magnetic properties can be regulated more easily (having well‐ordered AFM cores), leading to magnetic nanostructures with large coercivities, readily tunable blocking temperatures, and additionally improved magnetic effects.

#### 4.1.1 Physical Mechanism

**Figure 4:**
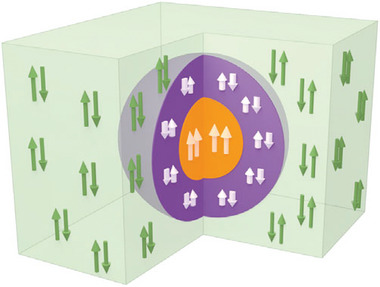
Schematic representation indicating the exchange bias effect between an FM core and an AFM shell. Most notably, the AFM anisotropy at the interface may be strong enough to increase the effective anisotropy of the FM core. Reproduced with permission.^[^ ^78^ ^]^ Copyright 2015, American Physical Society.

The term “exchange bias” describes unidirectional anisotropy, that is, a horizontal shift in the magnetization loop of systems containing a FM/AFM interface upon field‐cooling below the Néel temperature of the AFM component.^[^ ^76^ ^]^ The general consensus is that exchange bias arises from pinned magnetic moments within the AFM component at the interface, even though, in a representative system, only a limited proportion of the spins is actually pinned. The strength of the exchange bias at the FM/AFM interface can be assessed via the exchange bias shift (*H*_e_) and the enhanced coercivity (*H*_c_) from the FC *M*–*H* loops, which can be estimated by the expressions:^[^ ^77^ ^]^

(1) $$H_{e}=\left(H_{c1}+H_{c2}\right)/2$$

(2) $$H_{c}=-\left(H_{c1}-H_{c2}\right)/2$$

where *H*_c1_ and *H*_c2_ are the coercivities in the negative and positive field directions, respectively. For example, De Toro et al. recently presented a scheme of exchange‐bias particle stabilization that exploited magnetic proximity effects using inert‐gas condensation synthesized Co NPs embedded in RF‐sputtered NiO (**Figure** **4**).^[^ ^78^ ^]^ Although this example did not involve a *M*–Cr system, the principle it demonstrated was valid for this class of FM/AFM coupled NPs.

#### 4.1.2 Experimental Fabrication

Gas‐phase synthesis enables the design of mixed alloy and segregated core–shell NPs with independent control over core size and shell thickness; as such, it is an apt fabrication method to explore exchange couplings between different layers and the exchange bias effect.^[^ ^79^ ^]^ Research has focused on the evolution of the magnetic behavior of M‐NPs while being coated with a single or double monolayer of Cr. The objective was to identify the effect of the Cr‐shell thickness on the nature of the magnetic interface and detect the emergence of the exchange bias phenomenon.

Two major challenges are typically observed when fabricating FM/AFM structures. First, when metallic NPs are exposed to the environment, they readily form an oxide surface layer which affects the exchange properties and makes it particularly difficult to deconvolute the individual effects of Cr, Cr‐oxide, or *M*‐oxide. Second, element‐specific surface segregation of Cr atoms often occurs spontaneously. As a result, the majority of research focused on thin films with flat interfaces; the inspiration was the employment of such systems in spin valves used in read heads for magnetic recording applications. Therefore, despite the aforementioned original observations of exchange bias in NPs, relatively few studies on NP systems containing FM/AFM interfaces have been reported to date. There is still a dire need and plenty of room for more advanced studies that will take the use of gas‐phase synthesized *M*–Cr NPs from the lab to the industrial facility and into our houses as magnetic recording media.

##### Gas‐Phase Synthesis of FeCr NPs

**Figure 5:**
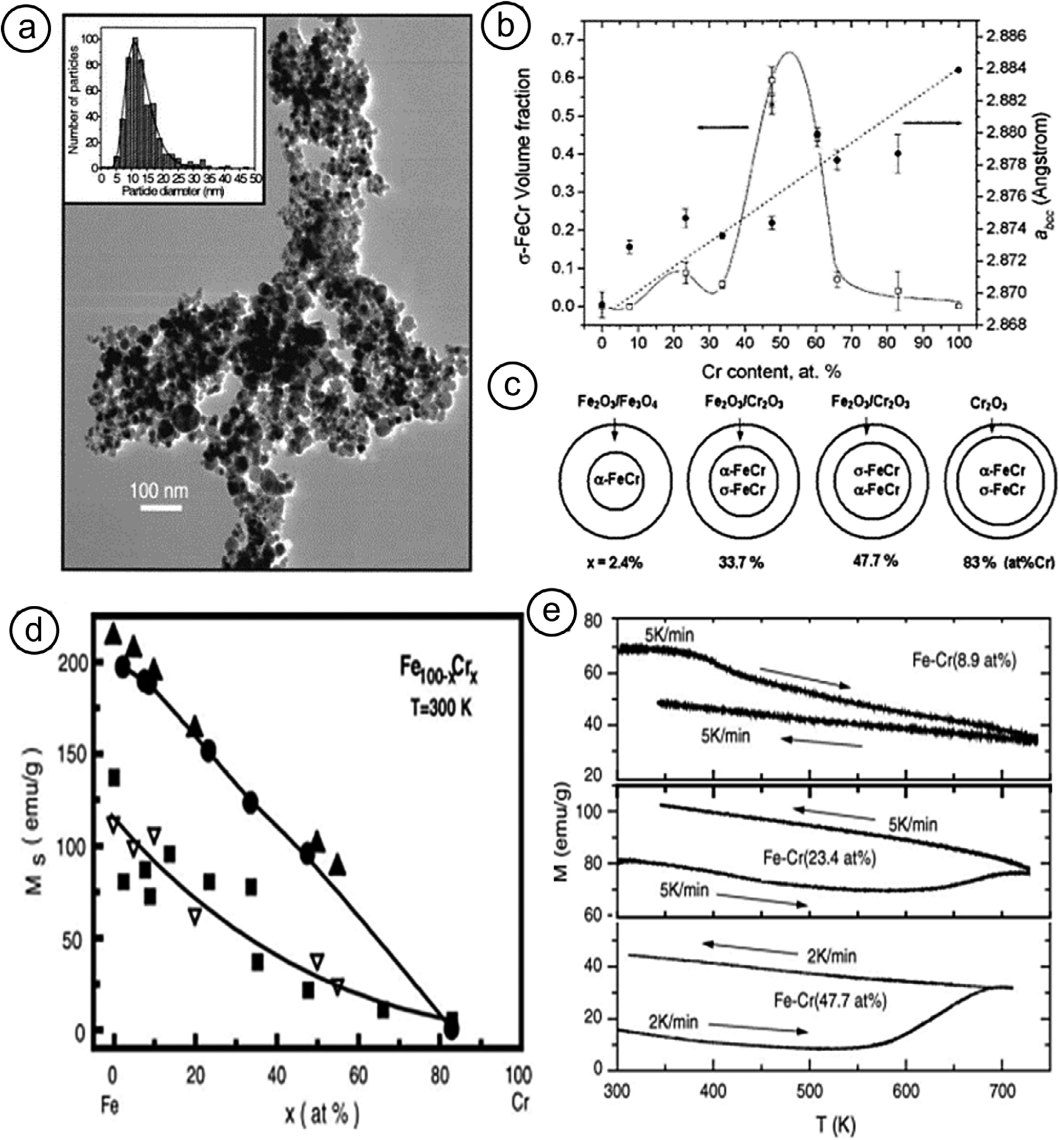
a) Transmission electron microscopy (TEM) image (and corresponding size‐distribution) of FeCr aerosol particles. b) Variation of σ‐phase volume fraction and lattice constant as a function of Cr content for the particles in (a). Reproduced with permission.^[^ ^57^ ^]^ Copyright 2005, AIP Publishing. c) Phase formation chart for various Cr at% concentrations. Reproduced with permission.^[^ ^27^ ^]^ Copyright 2008, Elsevier. d) Room temperature saturation magnetization, *M*_S_ versus chromium content, Cr at% for both NPs (closed square and open triangle) and bulk alloys (closed circle and closed triangle). e) Heating and cooling *M*–*T* curves of FeCr (8.9 at%, 23.4 at%, and 47.7 at%) NPs. Reproduced with permission.^[^ ^80^ ^]^ Copyright 2006, Elsevier.

Complex magnetic couplings were reported in gas‐evaporated Fe_1−_ *_x_* Cr*_x_* (0 < *x* < 0.83) aerosol particles (**Figure** **5**a).^[^ ^27^, ^57^ ^]^ The high temperature α‐FeCr (≈13 nm) and σ‐FeCr (≈10 nm) phases could both be stabilized at RT, while the former was the majority phase in all the examined compositions, save for the 47.7 at% and 60.4 at% Cr samples, where the latter phase constituted up to 60 wt% of the material (Figure 5b). The metastable σ‐FeCr phase was weakly magnetic, with an average magnetic moment of 0.1 µB per Fe atom and a *T*_C_ below 60 K. It was stable up to 550 K, whereas it started transforming to *bcc*‐FeCr when annealed at 700 K, additionally yielding Cr_2_O_3_ due to Cr surface segregation—a recurring feature we will also encounter below (Figure 5c). In the as‐prepared sample, the α‐FeCr particles (and their agglomerates) were separated by the PM σ‐FeCr phase, which generated SPM relaxation effects. Consecutively, the annealed sample, with a substantially reduced PM σ‐FeCr phase, was less sensitive to thermal fluctuations, and its magnetic character was dictated by the interparticle interactions. Both phenomena (namely, surface segregation and interparticle interactions) qualitatively differentiate the magnetic behavior of particulate samples from bulk ones.^[^ ^28^ ^]^

Room temperature saturation magnetization (*M*_S_) versus Cr concentration curves for both NPs and bulk alloys are shown in Figure 5d.^[^ ^81^ ^]^ Aerosol NPs show smaller *M*_S_ values than bulk alloys. At low Cr concentrations, the NPs only form the α‐FeCr phase, and a reduction of *M*_S_ thus emanates from the formation of an Fe‐oxide shell. With rising Cr concentration, a PM σ‐FeCr appears in NPs, which favors further reduction of *M*_S_.

Successive heating and cooling of the samples while measuring their magnetization as a function of temperature resulted in their structural transformation, which in turn affected their *M*(*T*) curves shown in Figure 5e. The gradual decrease of magnetization with increasing temperature, as observed for 8.9 at% Cr NPs, constituted typical FM behavior. Since subsequent cooling begun before the Curie point was reached, its leg along the magnetization curve lies beneath its warming counterpart; this can be due to at least partial recrystallization of the nanostructure. The lower magnetization values for 23.4 and 47.7 at% Cr particles at the first stage of heating are attributed to the presence of σ‐FeCr. Magnetization is minimized at ≈530 K and subsequently begins to rise; it eventually reaches a maximum at ≈700 K. This increase is attributed to the *σ* → *α* transition. During cooling, the *M*(*T*) curve rose smoothly with the same slope as that for 8.9 at% NPs, hence evidencing that it is governed by the α‐FeCr phase and the relevant occurrence of recrystallization at the surface layer. However, RT magnetization of the 23.4 at% Cr particles is appreciably higher than that of 47.7 at% Cr particles, evidencing the larger α‐FeCr content.

**Figure 6:**
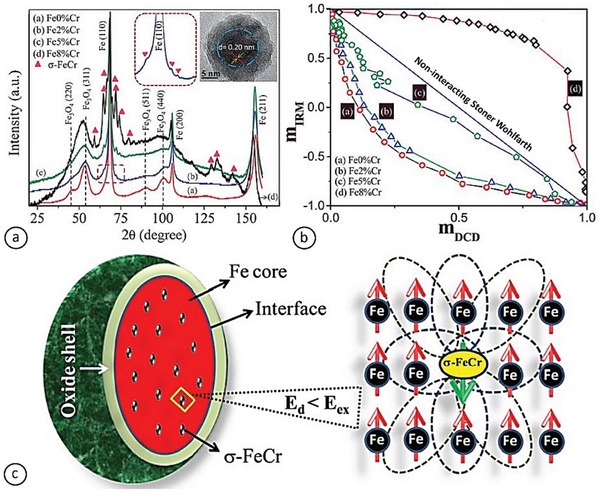
a) X‐ray diffraction pattern indicating the formation of the σ‐FeCr phase for FeCr NPs. b) Henkel plots *m*_d_(*H*) = *M*_d_(*H*) / *M*_d_(*H*_max_) versus *m*_r_(*H*) = *M*_r_(*H*) / *M*_r_(*H*_max_), which show the reversal of interaction from dipolar to exchange. Reproduced with permission.^[^ ^78^ ^]^ Copyright 2009, Royal Society of Chemistry. c) “Watermelon” model of Cr‐doped core–shell NP. The σ‐FeCr grain corresponds to the watermelon seed, which is exchange‐coupled with the surrounding *bcc*‐Fe (watermelon flesh). Reproduced with permission.^[^ ^82^ ^]^ Copyright 2013, AIP Publishing.

In another study, Cr‐doped core–shell Fe NPs (≈25 nm in diameter) containing 0 at%, 2 at%, 5 at%, and 8 at% Cr were grown via inert‐gas condensation.^[^ ^78^, ^82^ ^]^ Evidence of the formation of the σ‐FeCr phase and *bcc*‐Fe with merely 2 at% Cr doping is shown in **Figure** **6**a, which is unique because it had been reported that the σ‐phase forms above 20 at% Cr in NPs. The low concentrations of Cr (<10 at%) were selected so that complete conversions of the Fe‐oxide shell to Cr_2_O_3_ and of the Fe metal core to FeCr alloy were inhibited. The Cr content suppressed the saturation magnetization while increasing the number of σ‐FeCr seeds in the Fe‐core of Cr‐doped samples. Fierce competition was triggered between exchange and dipolar interactions by the exchange interaction between the *bcc*‐Fe and the *σ*‐FeCr grains. Dipolar to exchange interaction reversal was observed at 8 at% Cr. This was confirmed by the Henkel plot, as shown in Figure 6b, and explained through a theoretical “watermelon” core–shell model (Figure 6c), with σ‐phase precipitates uniformly spread throughout the Fe core resembling the watermelon seeds and flesh, respectively. There exist two positions in the NP where exchange interaction happens: i) at the interface between the Fe core and the Fe‐oxide shell (corresponding to the rind in the watermelon analogy) and ii) inside the core, between the seed (σ‐FeCr) and the flesh (Fe). The substantial contribution of interaction switching came from the σ‐FeCr–*bcc* Fe exchange interaction, since the interface interaction cannot compete with the dipolar interaction. Strong exchange coupling at the core contributed to the total interactions more significantly than the interparticle dipolar interactions, particularly in the Fe‐8%Cr. As a result, it became evident that the magnetic interactions in systems comprising core–shell FeCr NPs can be controlled by adapting the doping concentration of Cr.

##### Gas‐Phase Synthesis of CoCr NPs

Owing to their remarkable properties (e.g., high coercivity^[^ ^83^ ^]^), Co–Cr alloys are commonly considered for various applications such as magnetic recording media,^[^ ^83^ ^]^ ferromagnetic shape‐memory alloys,^[^ ^84^ ^]^ biomaterials,^[^ ^85^ ^]^ electronic devices,^[^ ^86^ ^]^ and communication equipment satellites.^[^ ^87^ ^]^ However, the majority of magnetic studies on the Co–Cr system focus on thin films with perpendicular anisotropy for applications in microwave sensing or magnetic recording.^[^ ^88^ ^]^ Fundamental studies on the magnetism of Co–Cr NPs are rather scarce; a few exemplar works are reviewed below.

**Figure 7:**
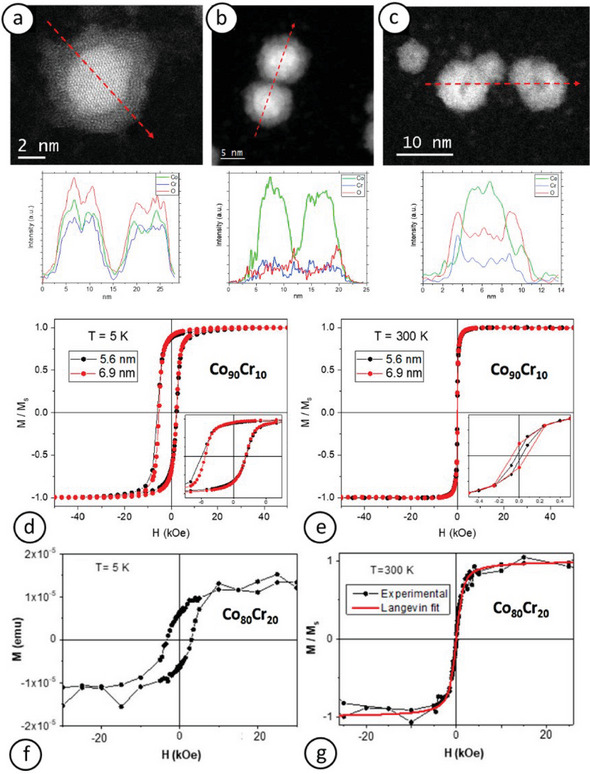
EELS images and corresponding elemental profiles extracted along the red dash arrows of exemplar NPs from the: a) Co_90_Cr_10_ (5.6 nm in diameter), b) Co_90_Cr_10_ (6.9 nm in diameter), and c) Co_80_Cr_20_ (7 nm in diameter) samples. d–g) *M*–*H* curves at 5K (d,f) and 300 K (e,g) for Co_90_Cr_10_ (5.6 and 6.9 nm in diameter) (d,e) and Co_80_Cr_20_ (7 nm in diameter) (f,g). Reproduced with permission.^[^ ^88^ ^]^ Copyright 2020, MDPI.

Soler–Morala et al.^[^ ^88^ ^]^ investigated the effects of particle size and Co/Cr ratio on the properties of NPs synthesized using a magnetron‐based inert‐gas condensation source from two alloy targets with Co_90_Cr_10_ and Co_80_Cr_20_ nominal compositions. The point at which a segregated Co‐core@Cr‐shell structure, accompanied by inevitable oxide formation, spontaneously forms was correlated with the particles' surface‐to‐volume ratio (**Figure** **7**). When this ratio was higher than 1 (i.e., for particles 5.6 nm in diameter, Figure 7a), a full shell could not be formed, whereas when this ratio decreased below 1 (i.e., for particles ≈6.9 nm in diameter, Figure 7b), the proportion of surface segregated Cr atoms sufficed for the formation of a complete shell. When the Cr proportion in the target was increased to Co_80_Cr_20_, it resulted in thicker shells spontaneously formed for similar particle sizes (i.e., ≈7 nm in diameter, Figure 7c).

The influence of the composition and structure of these core–shell particles on their magnetic response is evaluated in Figure 7d–g. Using a Co_90_Cr_10_ target, FM NPs stable over a broad temperature range below 300 K were synthesized; thus, significantly lowering the critical size for RT superparamagnetism to well below 7 nm. This reduction stemmed from increased total anisotropy due to exchange bias generated by the coupling between different magnetic phases in the core and shell. Smaller particles (5.6 nm in diameter) exhibited more pronounced interfaces, leading to higher coercive field and exchange bias. Combined with dipolar interactions between particles, this contributed to their hard magnetism at low temperatures and blocked response at RT. Even though particles with a higher Cr proportion remained SPM at RT, they exhibited nearly twice the anisotropy of conventional *fcc* Co nanoparticles.^[^ ^88^ ^]^

In a different study, Lee et al. fabricated arrays of CoCr binary nanocluster wires (NCWs).^[^ ^89^ ^]^ These NCWs, varying in diameter from 10 to 20 nm, aggregated into bundles reaching lengths of 2–3 mm, which, in turn, formed a 3D array. CoCr NCWs containing 0.8–2.2 mol% Cr exhibited an exclusive Co‐rich phase and demonstrated enhanced magnetic properties. Specifically, they displayed nearly double the coercivity compared with arrays composed of pure Co NCWs, along with high values of *M*_r_/*M*_S_ (where *M*_r_ represents magnetization remanence). Co‐rich phase domains could be magnetically isolated from each other by a nonmagnetic Cr‐rich phase when the Cr mol content was more than ≈3%. The remarkable magnetic properties of the Co‐rich phase suggest potential applications for these arrays in high‐density perpendicular magnetic recording, pending further technical refinements.

#### 4.1.3 Application Potential

The emergence of giant magnetoresistance (GMR)^[^ ^90^ ^]^ marked a significant shift in research focus from bulk alloy materials to nanostructures of *M*–Cr.^[^ ^90^, ^91^ ^]^ GMR is a quantum‐mechanical effect that emerges from AFM exchange coupling between two magnetic layers separated by a non‐magnetic spacer, as seen in the *M*/Cr/M multilayer system. This effect attracted considerable interest in combining *M*(Fe, Co, Ni) with Cr to explore their properties. At interfaces, additional important phenomena occurred, such as induced magnetic moments in atoms of the typically non‐magnetic Cr metal (with a Néel temperature, *T*_N_, of 311 K) when in proximity to magnetic metals. This effect facilitated the development of highly sensitive magnetic field sensors for magnetic hard disk read heads, thereby revolutionizing magnetic recording.^[^ ^92^ ^]^

A key driver in spintronics and magnetic device applications is the persistent demand for the miniaturization of device components. This motivation has led to the proposition of magnetic NPs as potential candidates to significantly increase storage/area in magnetic random‐access memory (MRAM) devices and hard‐disk drives due to their diminutive size. Particularly promising are AFM‐based NPs that incorporate FM/AFM components, offering tunable magnetic properties. This includes the ability to surpass the superparamagnetic limit, that is, tune the blocking temperature (*T*_B_), above which the NPs collectively contribute to a superparamagnetic state beyond RT. Such nanostructures offer several advantages over larger FM/AFM‐based systems, including weak inter‐particle interactions, semi‐metal properties (i.e., spin‐dependent switching between insulating and conducting states), and faster switching times between states. Despite significant progress, challenges remain, such as synthesizing stable patterned assemblies of magnetic core–shell NPs and achieving sufficiently rapid switching times between spin states.^[^ ^70^, ^93^ ^]^

### 4.2 Curie Temperature Tuning for Magnetic Hyperthermia

Incidence of cancerous disease has often been projected to increase worldwide in the near future (e.g., by more than 75% by the year 2030, according to a recent study^[^ ^94^ ^]^). Therefore, a primary challenge in biomedical research involves discovering effective new therapies or mitigating the adverse side effects of existing treatments. In addition to conventional medical approaches such as chemotherapy and radiotherapy, thermotherapy has emerged as a promising treatment option. Cancer cells are more vulnerable to heat than normal cells, making thermotherapy a viable strategy. Unlike healthy cells, tumor cells experience limited heat dissipation when heated to temperatures between 41 °C and 47 °C because their tightly packed structure restricts blood vessel dilation and circulation. Harmful metabolic by‐products and low pH are thus accumulated, driving the abnormal growth to self‐destruction.^[^ ^95^, ^96^ ^]^

Curie temperature is the temperature at which a material undergoes a FM‐to‐PM transition (or vice versa). The possibility to tailor the *T*_C_ in *M*–Cr nanoalloys (via controlling the Cr content) allows for the optimization of magnetic parameters such as the saturation magnetization (*M*_S_) and coercivity; as such, it presents a notable advantage for various applications. The most well‐known example concerns the heat generated through hysteresis losses when NPs are exposed to an alternating magnetic field (AMF), which can be used for targeted cancer therapy. The tunability of the *T*_C_ ensures that the NPs achieve their maximum heating effect within the therapeutic temperature range. This improves their utilization safety because it acts as a “safety switch” that turns off to prevent potential overheating and its resultant excessive tissue damage.

#### 4.2.1 Physical Property

In magnetic hyperthermia, the attained local heating is based on remagnetization losses. When magnetic thermo‐seeds or magnetic NPs with *T*_C_ ≈42–45 °C, high heating efficiency, and biocompatible properties are subjected to AMFs, the body temperature is locally heated up.^[^ ^96^ ^]^ Due to their small size, NPs can be considered single‐domain; this means that the heating stems from the movement of the particles themselves (Brownian relaxation) or from the magnetic moment rotation (Néel relaxation).^[^ ^59^ ^]^ In reality, both mechanisms may occur concurrently, and the amount of heat generated depends on their relative contributions.^[^ ^97^ ^]^ The method allows for controllable heat dissipation, and as a result, side effects are markedly less severe than in conventional hyperthermia treatments.

The heating capability of magnetic NPs is typically quantified by the specific absorption rate (SAR), which correlates with the power absorbed by a volume of biological tissue when exposed to external alternating magnetic fields (AMFs):

(3) $$\mathrm{SAR}\;=\frac{C}{m}\frac{dT}{dt}$$

where *C* is the heat capacity of the medium, *m* the total mass of magnetic materials, and d*T*/d*t* the heating rate.^[^ ^97^ ^]^ The specific loss power (SLP) is often used interchangeably with SAR, measuring the rate of energy dissipation per unit mass of the magnetic NPs for a given frequency during heating experiments conducted at this specific frequency.^[^ ^80^, ^98^ ^]^

High SAR values are crucial for minimizing hyperthermia agent dosage and exposure time. Studies show that SAR values depend on crystallite size.^[^ ^98^, ^99^, ^100^, ^101^, ^102^ ^]^ Shape is even more important as it is related to anisotropy, with, for example, cubic‐shaped NPs exhibiting higher SAR than spherical ones^[^ ^103^ ^]^ (for instance, simulation results suggest that cubic Zn_0.4_Fe_2.6_O_4_ NPs with surface‐disordered spins show enhanced SAR by 4% compared with spherical NPs^[^ ^104^ ^]^). Finally, surface coating, sometimes necessary to overcome biocompatibility or toxicity issues, may affect Brownian relaxation and heat transfer, leading to varied SAR values.^[^ ^105^ ^]^ Considering this, thanks to their high saturation magnetization and, most importantly, tunable *T*_C_ (though often at the cost of lower SAR values compared with ferrite‐based NPs), compounds of bimetallic magnetic nanostructures such as NiCr and FeCr appear attractive.^[^ ^106^, ^107^, ^108^, ^109^, ^110^ ^]^ However, their customizability ensures that the SAR remains within safe and effective limits, thus minimizing the risk of damaging healthy tissues while maximizing the efficacy of cancer treatment.

#### 4.2.2 Experimental Fabrication

Studies on NiCr NPs fabricated by various physical and chemical methods identified distinct magnetic properties.^[^ ^111^ ^]^ For example, Ban et al. investigated the heating effect of solid Ni_72_Cr_28_ powder fabricated via microemulsion and mechanical milling, finding that the self‐heating temperature during hyperthermia experiments exceeded the targeted Curie point significantly due to heterogeneities in particle sizes and composition.^[^ ^112^ ^]^ This heterogeneity (and its resultant effects) was inherent to the fabrication method, once more underlining the potential merits of gas‐phase synthesis with its capabilities for configurational control and in‐flight mass filtration. Interestingly, RT ferromagnetism was reported even in NiCr particles containing 29 wt% of Cr. In other works, the *T*_C_ of the Ni_1−_ *_x_* Cu*_x_* ^[^ ^113^ ^]^ and γ‐FeNi^[^ ^110^ ^]^ NPs was effectively lowered and maintained within the recommended therapeutic range, albeit at a cost of decreased saturation magnetization.

##### Gas‐Phase Synthesized NiCr NPs

**Figure 8:**
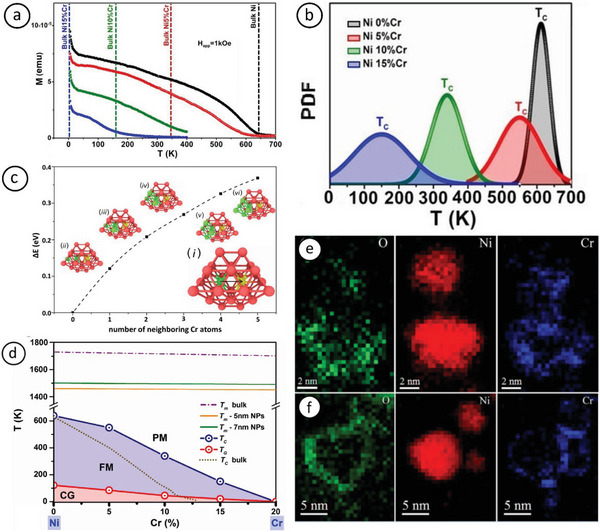
a) Field‐cooled (FC) magnetizations versus temperature under a magnetic field of 1 kOe across the temperature range of 5 ≤ *T* ≤ 700 K, with *T*_C_ values of bulk Ni_1−_ *_x_* Cr*_x_* alloys also provided for comparison. b) Probability distribution functions of *T*_C_ obtained from normalized FC magnetization versus temperature graphs under 1 kOe, illustrating a clear shift toward lower values with increasing Cr concentration. c) 1NN vacancy migration considering the atomic local environment, also including second‐nearest‐neighbor (2NN) atoms. Green, red, and yellow colors denote Cr atoms, Ni atoms, and vacancy sites, respectively. The migration path is depicted in inset (c‐i), with configurations in insets (c‐ii–vi), showing increasing sizes of neighboring Cr‐atom precipitates (from one to five atoms). d) Magnetic phase diagram (Ni‐rich region), showing size‐dependent depression of melting points calculated from molecular dynamics (MD) caloric curves. Blue and red open circles indicate Curie (*T*_C_) and cluster‐glass (*T*_G_) temperatures, respectively. The violet region represents the long‐range FM order below *T*_C_, extending over a larger area compared with bulk data (indicated by the black dotted line) due to Cr segregation. The pink region below *T*_G_ signifies the transition from the FM state to the cluster‐glass state, observed at very low temperatures. Reproduced with permission.^[^ ^68^ ^]^ Copyright 2017, American Physical Society. Electron energy loss spectroscopy (EELS) images of NiCr nanoparticles with 5 at% Cr: e) before and f) after annealing, clearly illustrating both extensive Cr surface segregation and oxide‐shell formation, even in the as‐grown samples. Reproduced with permission.^[^ ^111^ ^]^ Copyright 2015, American Chemical Society.

Recently, NiCr (0–15 at% Cr) NPs ≈5 nm in diameter were fabricated by CBD,^[^ ^68^ ^]^ showing markedly higher *T*_C_ values than their parent bulk alloys (**Figure** **8**a). Normally, *M*–Cr NPs exhibit low *T*_C_ values and saturation magnetization; this stems from the intricate interplay between finite size effects, associated with the reduced number of exchange‐coupled spins inside the NP cores, and surface effects, associated with the local symmetry breaking in the lattice, defects, dangling bonds, and surface oxidation.^[^ ^114^ ^]^ However, the one‐step gas‐phase synthesis method enabled tuning the *T*_C_ within a broad temperature range, as required for magnetic hyperthermia application. What is of particular importance is that this was achievable by merely controlling a single parameter, that is, the concentration of Cr in the NPs (Figure 8b).

This was attributed to the surface segregation phenomenon, also prevalent in the Ni–Cr system,^[^ ^115^ ^]^ as was in the Fe–Cr case mentioned above (Section 2.1.2). In situ annealing measurements up to 300–800 K conducted under vacuum by aberration‐corrected environmental transmission electron microscopy (E‐TEM) (Figure 8e,f) and vibrating sample magnetometry (VSM) showed gradual Cr segregation occurring with increasing temperatures, which, at 700 K, culminated in the formation of core‐satellite structures.^[^ ^111^ ^]^ However, overcompensation of just 5 at% Cr (compared with bulk compositions) yielded similar *T*_C_ values to their bulk counterparts. While complete surface segregation of Cr was anticipated up to this concentration, it was theoretically shown that residual Cr remained inside particle cores for doping levels > 5 at%.^[^ ^68^ ^]^ The explanation is based on the markedly higher activation barrier for the diffusion of small Cr precipitates compared with that of single Cr atoms, combined with the probability of finding such precipitates as a function of overall Cr concentration in the *fcc* Ni matrix (Figure 8c). This was summarized in the nanophase diagram shown in Figure 8d, indicating both structural and magnetic characteristics. The ensuing competition between FM and AFM ordering generated a diverse array of intriguing phenomena, including a cluster‐glass ground state at very low temperatures and enhanced *T*_C_ values.

**Figure 9:**
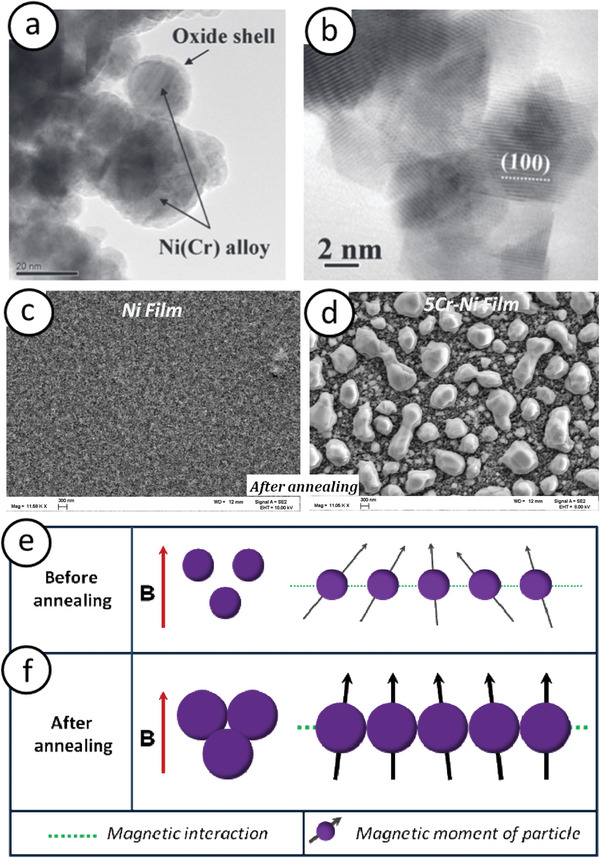
TEM images of a) Core–shell and b) fully oxidized Ni‐5Cr particles. Reproduced with permission.^[^ ^117^ ^]^ Copyright 2011, American Scientific Publishers. SEM images of granular Ni and Ni‐5Cr films on a Si substrate: c) prior to and d) post annealing. e,f) Schematic depiction illustrating the augmentation of magnetic interactions and magnetic moments among particles due to particle growth and aggregation induced by annealing. Adapted with permission.^[^ ^116^ ^]^ Copyright 2011, IEEE.

A comprehensive picture of the structural evolution of NiCr nanoalloys under thermal treatment in ambient conditions was provided by Sundararajan et al., who synthesized low concentration (< 10 at%) Cr‐doped NiCr NPs using a nanocluster deposition system; their goal was to study any structural, physical, and/or magnetic property modifications induced by heat treatment (HT).^[^ ^116^, ^117^ ^]^ This study provided a variety of details about air passivated pure‐Ni and Cr‐doped Ni NPs with sizes ranging from several nm to several tens of nm. Both pure‐Ni and Cr‐doped Ni particles possessed core–shell structures comprising a metal core covered by an oxide layer, of a typical thickness of ≈1.6 nm. It was found that, below a critical size of ≈6 nm, the particles were fully oxidized into NiO (**Figure** **9**a,b). Cavities were also observed in the fully oxidized particles owing to an outward diffusion of Ni through the oxide (Kirkendall effect^[^ ^118^ ^]^). Above this critical size, particles formed a yolk–shell structure with the presence of voids beneath the shell.^[^ ^119^ ^]^

However, after a 30‐min heat treatment at 600 °C under constant Ar gas flow, the average size of both pure‐Ni and Ni‐5Cr NPs increased from 20 to 50 nm (Figure 9c,d). The *M*_S_ increase was more pronounced for the Ni‐5Cr particles than for the Ni film. This was attributed to the rapid aggregation and resultant growth of nanoparticulated Ni‐5Cr films owing to the presence of Cr, which acted as a catalyst for particle growth and aggregation. Consequently, this led to an increase in magnetic interaction (Figure 9e,f) and magnetic moments of the particles, resulting in enhanced magnetization of the annealed granular films.

These results signify the importance of sample homogeneity for the production of predictable (and hence, reproducible) magnetic properties in *M*–Cr nanoalloys, emphasizing the sensitive dependence of nanostructure (and resultant properties) on the details of the fabrication method.

#### 4.2.3 Application Potential

**Figure 10:**
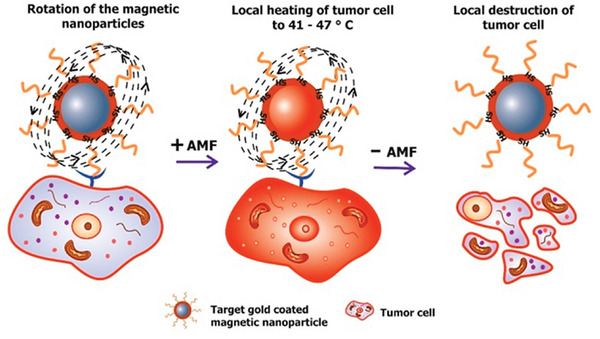
Schematic illustration of magnetic NP‐mediated hyperthermia treatment for selective tumor cell destruction. Reproduced with permission.^[^ ^120^ ^]^ Copyright 2017, MDPI.

Hyperthermia‐level temperatures can be induced using AC electromagnetic fields, ultrasound, heat radiation, perfusion, or microwave radiation, depending on the depth and specific location of the target site.^[^ ^120^ ^]^ Conventional hyperthermia treatments are typically performed by immersing the patient's body in a hot water bath (general hyperthermia) or by applying radio‐frequency (RF) and microwave radiation (local hyperthermia). However, conventional hyperthermia methods trigger various side effects as a result of the uneven heat dissipation inside the tumor and healthy tissue. To confront this challenge, significant research focuses on the possibility of directing and localizing magnetically controlled thermal energy. This aims to enhance therapeutic effectiveness at specific depths within the body while mitigating undesired heating side effects;^[^ ^121^ ^]^ enter NP‐based magnetic hyperthermia (**Figure** **10**).^[^ ^3^, ^120^ ^]^

From the viewpoint of biomedical applications, biological or chemical modifications are required to meet requirements regarding biocompatibility, or site‐specific delivery; for example, coating and post‐growth functionalization of aforementioned magnetic NPs with oleic acid^[^ ^100^ ^]^ or polyethylene glycol (PEG)^[^ ^122^ ^]^ reduces their cytotoxicity, rendering them suitable candidates for self‐regulated magnetic hyperthermia and other biomedical applications (However, it should be emphasized that no metal‐based NPs (*M*–Cr included) have yet been approved by the US Food and Drug Administration (FDA) or the European Medicines Agency (EMA); comprehensive lists of, mostly lipid‐based or polymeric, approved NPs can be found here^[^ ^123^ ^]^ and here.^[^ ^124^ ^]^ In general, NPs are subject to the same regulatory requirements as other chemical substances (for example, under Registration, Evaluation, Authorisation and Restrictions of Chemicals ‐REACH‐ in the European Union).^[^ ^125^ ^]^ To date, however, there is no single globally adopted technical definition applicable to all nanomedicines; in fact, nanomedicine classification is challenging as there are two applicable regulatory frameworks with significantly different requirements for the two domains. NPs for biomedical applications can be classified either as medicines or medical devices; to complicate things further, this classification is not always consistent across global regulators. This means that what could be classed as a medicine in one country could be considered a medical device in another, with the resultant differentiation in specific safety and efficacy standards that must be adhered to. Finally, assuming that a *M*–Cr NP based hyperthermia treatment is classified as a medicine in a specific area of jurisdiction, it will need around 12 years to reach the market after successfully completing all clinical trials required by the corresponding responsible body that can grant authorisation.^[^ ^126^ ^]^). By application of nonmagnetic or magnetic ions with low *T*_C_ (e.g., Cr), one can reduce the exchange interaction constant between the magnetic ions in the NPs, which leads to lower *T*_C_ values. Ni‐based alloys can be utilized as thermoseeds by adjusting their Curie points for achieving thermal self‐regulation; thereby, eliminating the expensive and invasive thermometry currently accompanying hyperthermia treatments.

### 4.3 Magnetocaloric Effect for Magnetic Cooling

The usage of air conditioning and refrigeration accounts for roughly 17% of worldwide electricity consumption,^[^ ^127^ ^]^ equivalent to ≈2000 TWh. The International Energy Agency (IEA) predicts that unless the efficiency of these systems is enhanced or new technologies are employed, this amount could rise to 37% by 2050.^[^ ^128^ ^]^ Until now, the majority of refrigeration, air‐conditioning, and heat pumping systems have relied on mature vapor‐compression technology, which boasts minimized production and maintenance costs, and safe and reliable operation. This technology also provides relatively high second‐law energy efficiency of up to 60%^[^ ^129^ ^]^ for large‐scale appliances. However, for small‐scale appliances, the efficiency based on the Carnot cycle is usually considerably lower, ≈20%.^[^ ^130^ ^]^ Typical cooling technologies are based on refrigerant gases including hydrofluorocarbons, which can be directly emitted to the environment by leaks during operation or maintenance and at the end of the device lifespans. These emissions comprise 7.8% of the total global greenhouse gas emissions^[^ ^127^, ^131^ ^]^ and are expected to be phased out by initiatives such as the Kigali amendment within the next few decades as a means of curbing climate change;^[^ ^132^, ^133^ ^]^ clearly, alternative cooling technologies are in dire need.

Magnetic cooling (or refrigeration) is such a technology. Although not a new idea (it was first proposed and implemented almost a century ago, in particular regarding applications at extremely low temperatures), it currently gains traction thanks to its environmental friendliness due to the use of solid substances as working materials. Most importantly, with respect to this review article, its high efficiency brings it to the forefront in view of the rapid developments in portable battery operated apparatuses and miniaturized devices; hence, the renewed interest in nano‐magnetic refrigeration.

#### 4.3.1 Physical Mechanism

The physical mechanism behind magnetic cooling is the magnetocaloric effect, defined as a change in the temperature of a material when exposed to an external alternating magnetic field.^[^ ^134^ ^]^ The magnetocaloric effect can be boosted through a significant variation in magnetic field, the use of magnetic materials with low heat capacity, or magnets exhibiting substantial changes in net magnetization relative to temperature under constant magnetic field conditions. The caloric effect is reversible, whereas vapor refrigerants are compressed or expanded irreversibly. Caloric materials are solid; thus, they are not threatening for the environment in the same manner that other refrigerants are. Magnetocaloric cooling is the most developed among all the other caloric‐based technologies (such as electrocaloric or mechanocaloric). Theoretical simulations have been extensively employed to elucidate the underlying physical mechanisms and to optimize materials with enhanced magnetocaloric properties, for example, refs. [135, 136, 137, 138, 139]. Such simulations studied the temperature dependence of the magnetocaloric effect in alloys under investigation, as well as changes in their magnetic entropy and adiabatic temperature, and, in many cases, the results helped elucidate the factors that contribute to the magnetocaloric effect.

A relevant performance metric is relative cooling power (RCP), defined as the product of the maximum change in entropy (∆*S*_M_) and the full‐width‐at‐half‐maximum (δ*T*_FWHM_) of the entropy versus temperature curve:

(4) $$\mathrm{RCP}=\Delta S_{M}\times \delta T_{\mathrm{FWHM}}$$

Thus, the effect of a giant working temperature range (δ*T*_FWHM_) can be beneficial in boosting the RCP. A way to improve this is by broadening the Δ*S*_M_ peak around the magnetic transition temperature.^[^ ^140^, ^141^, ^142^ ^]^ The magnetic entropy change (Δ*S*_M_) of a magnetic system under adiabatic magnetic field variation from 0 to final value *H*_max_ can be calculated using Maxwell's relation of thermodynamics:

(5) $$\Delta S_{M}=\int_{0}^{H_{\max}}{\left(\frac{\partial M}{\partial T}\right)}_{H}\;dH$$

FM‐AFM NPs often present such a possibility due to their distributed exchange coupling.

#### 4.3.2 Experimental Fabrication

Chaudhary and Ramanujan reported the fabrication via high‐energy planetary ball milling of *M*–Cr NPs (where *M* = Fe‐Ni) dispersed in oleic acid, thus comprising a ferrofluid employed as a heat transfer medium in a self‐pumping magnetic refrigeration prototype device.^[^ ^143^ ^]^ They confirmed the aforementioned possibility of NPs to modify the Δ*S*_M_ and corresponding RCP as well as the *T*_C_ by varying the Cr content, due to the AFM character of Cr, and compared with other systems in powder or coarser forms fabricated with a variety of methods. In a more recent study, Radhakrishnan et al. fabricated CoCr*_x_* FeNi alloys by additive manufacturing (specifically, laser‐directed energy deposition, L‐DED).^[^ ^144^ ^]^ In this case *M* (in *M*–Cr) was not a single element, Co, Fe, or Ni, but rather a combination of them, forming a concentrated solid solution alloy when mixed with Cr. Even though in this case the alloys were bulk, pre‐alloyed powders of CoFeNi and CoCrFeNi were used as feed material. They also observed a linear decrease of *T*_C_ and monotonical decrease of *S*_M_ with increasing Cr content. The alloys displayed soft‐magnetic behavior (i.e., low coercivity) which was, however, insensitive to Cr concentration. Therefore, both studies confirmed that the magnetic behavior of the FM alloy could be systematically adjusted by adding AFM Cr.

##### Gas‐Phase Synthesized NiCr NPs

Very recently, Bohra et al.^[^ ^68^ ^]^ tried on different theoretical models to identify which best corresponded to the magnetic behavior of their CBD‐grown NiCr NP samples; they concluded that, for dense systems, it is a Gaussian Distribution model that reproduces the magnetic measurements (*M*–*T* curves) more accurately.^[^ ^145^ ^]^ Interestingly, this observation suggests that the intrinsic characteristics of the model align with the physical attributes of their samples (*T*_C_ distribution, NP size, structure and distance inhomogeneities, etc.).

In contrast, a hyperbolic tangent model was found better suited for thin films, which only display a single *T*_C_ value. Most importantly, they showed that the *T*_C_ distribution of the nanoparticulated samples has a side‐effect on the relative cooling power of the samples, which may be useful for magnetocaloric effect related applications.

**Figure 11:**
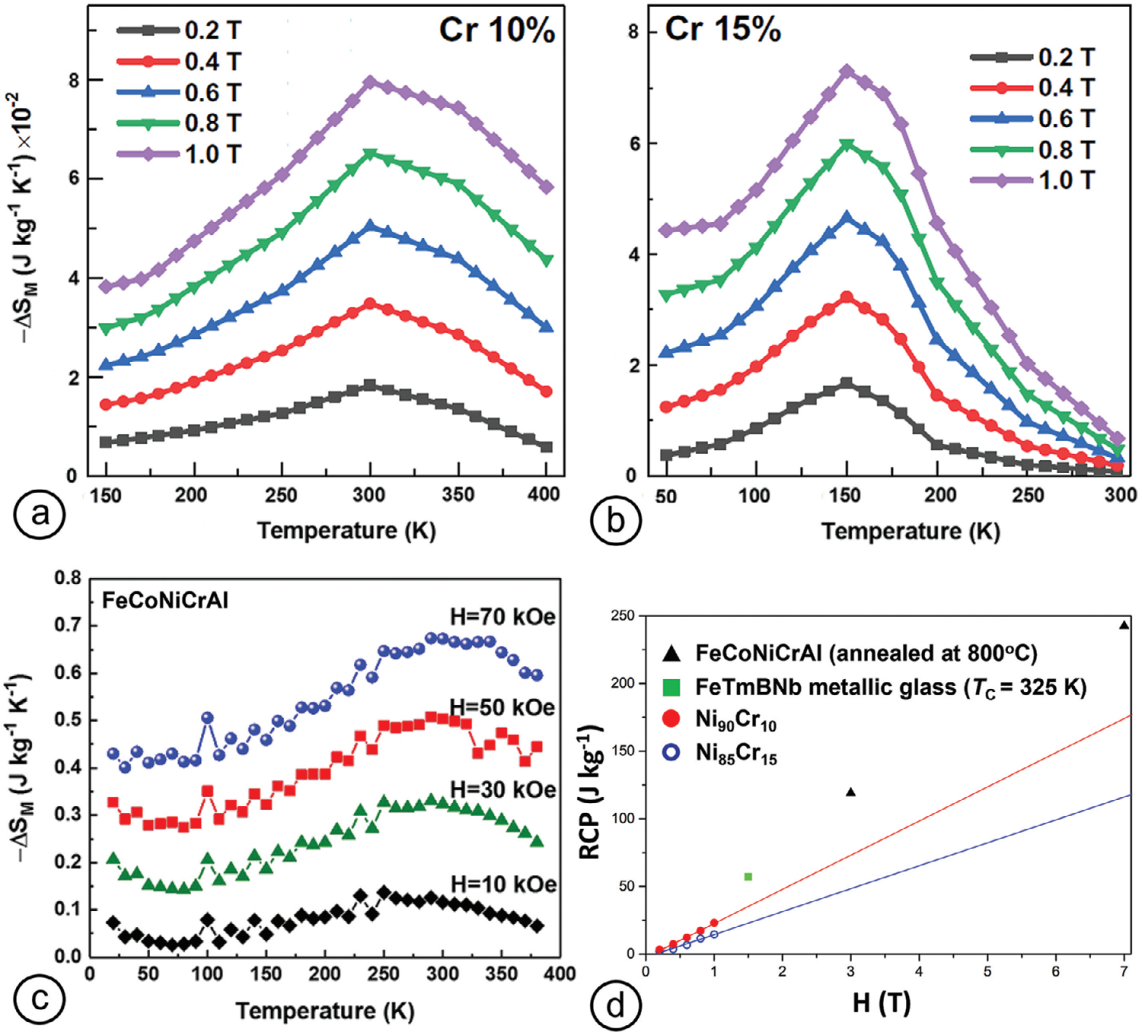
Temperature dependence of the change in magnetic entropy for a) NiCr 10 at% Cr thin film and b) NiCr 15 at% Cr thin film. Reproduced with permission.^[^ ^147^ ^]^ Copyright 2023, Springer Nature. c) FeCoNiCrAl (annealed at 800 °C). Reproduced with permission.^[^ ^148^ ^]^ Copyright 2019, AIP Publishing. d) RCP of samples from (a–c) as well as from a FeTmBNb metallic glass sample with relevant *T*_C_. The RCP values from NiCr samples were measured up to 1 T, but, for comparison, were linearly extrapolated toward higher *H* values, indicating relatively comparable RCPs (especially for 15 at% Cr). Plotting for this review was done from the data points in ref. [149].

Previously, Mathew and Kaul had utilized inert‐gas condensation fabricated Gd polycrystalline samples with grains in the nm size range to demonstrate the usage of nano‐crystallite size distribution as control parameter to maximize the magnetocaloric effect.^[^ ^146^ ^]^ Very recently, NiCr (0–15 at% Cr) nanoparticulated films were deposited by CBD (e.g., **Figure** **11**a,b).^[^ ^147^ ^]^ Changes in Cr concentration had a practically negligible effect on Δ*S*_m_ values. However, they affected magnetization and transition temperatures dramatically, moving the Δ*S*_m_ peaks toward RT values for 10 at% Cr. RCP values increased linearly with applied magnetic field (0–1 T), reaching values of 23.06 and 14.6 J kg^−1^ for 10 and 15 at% Cr, respectively. Linear extrapolations of RCP values for higher magnetic fields are shown in Figure 11d, along with respective values obtained from FeCoNiCrAl HEAs prepared by arc melting^[^ ^148^ ^]^ and FeTmBNb metallic glasses,^[^ ^149^ ^]^ implying that NiCr nanoparticulated films did not fare too badly, compared with the bulk alloys (Δ*S*_m_ values for the FeCoNiCrAl HEA are shown in Figure 11c).

It is worth mentioning here that HEAs are a relatively new class of alloys formed by equiatomic (or nearly equiatomic) mixing of five or more elements,^[^ ^150^, ^151^ ^]^ including *M*–Cr (where M is a combination of elements);^[^ ^152^ ^]^ medium‐entropy alloys containing fewer elements are also of interest because their crystallographic structure may depend on magnetic frustration as discussed above.^[^ ^153^, ^154^ ^]^ HEAs are chemically disordered (hence, high‐entropy alloys) but physically ordered; in that sense, they are different from metallic glasses which are chemically ordered but physically disordered. HEAs have recently been explored in depth due to their superior structural properties and corrosion resistance. Most interestingly, they can be engineered in nanoparticulate form^[^ ^155^, ^156^, ^157^ ^]^ or in thin‐films by magnetron‐sputtering.^[^ ^158^ ^]^ A combined approach would make them superb candidates for magnetic cooling applications, especially for small‐scale appliances.

#### 4.3.3 Application Potential

In addition to the overall challenges associated with magnetic cooling systems (such as the requirement for magnetic source shielding, high costs of magnetic field sources, and limited temperature range per cooling cycle in permanent magnet systems),^[^ ^159^ ^]^ materials‐related considerations are important. To start with, functional materials in magnetic refrigerators have to display a reversible phase transition during operation.^[^ ^160^ ^]^ Currently, typical candidate materials either incorporate costly rare‐earth (e.g., LaFeSi) or toxic (MnAsSb) elements, or exhibit inadequate mechanical properties (Heusler alloys), hindering their application in commercial magnetic refrigerators. Therefore, the search for new magnetocaloric materials demands that the material not only exhibit desirable magnetocaloric properties (such as a wide temperature range and low hysteresis losses) but also meet the typical requirements for household devices, including good corrosion resistance, mechanical properties, and non‐toxicity.^[^ ^161^ ^]^ Rare‐earth based materials, such as *R*_5_ *T*_4_ (where R  =  rare‐earth and T  =  transition metal) compounds, demonstrate giant magnetocaloric effect and corresponding potential for refrigeration applications.^[^ ^162^, ^163^ ^]^ However, transition‐metal based materials (including *M*–Cr) are generally preferable on grounds of raw materials supply and cost.^[^ ^141^, ^160^ ^]^ This is mostly due to the fact that rare earths supply chains are associated with monopolistic practices, price fluctuations, and supply risk due to geopolitical issues, along with environmental hazards during their mining, separation, and processing.^[^ ^161^, ^162^, ^163^, ^164^, ^165^, ^166^, ^167^ ^]^ Cost analysis has concluded that currently the focus is shifted from improving the sheer refrigeration capacity toward the discovery of less‐critical materials, while maintaining as optimal a refrigerating performance as possible.^[^ ^141^ ^]^

## 5 Computational Methods and their Application for *M*–Cr Compounds

Description of atomistic systems is quite complex due to an interplay of different phenomena at different scales. This often makes analytical mathematical modeling challenging as unavoidable oversimplistic approximations may limit models to account only for particular interactions of specific systems under certain conditions. This complexity becomes even more apparent for the treatment of magnetic systems. On the other hand, the exponential increase of computational processing power available in high‐performance computing (HPC) infrastructure, along with the widespread use and development of a variety of high‐quality, well‐established, open source or commercial software, renders in silico experiments the preferable route for efficiently obtaining accurate theoretical insights.

As the interpretation of observed phenomena at the nanoscale, and consequently the understanding of the impact of microscopic effects to the properties of *M*–Cr NPs are among the goals of this review, this section focuses on the role of electronic structure and atomistic simulations toward this direction.

### 5.1 Ab Initio Methods

Ab initio simulations constitute a family of methods which, in broad strokes, are based on quantum mechanical formulations to provide information about the electronic structure of a given system. Consequently, this description can be used to further assess structural, magnetic, thermal, optical, and other properties of the simulated system. Density functional theory (DFT) based on the Kohn–Sham approach is among the most popular ab initio methods in the scientific community, with a bibliometric study reporting a doubling of publications employing DFT every 5 to 6 years;^[^ ^168^ ^]^ therefore, various applications of DFT methods for exploring *M*–Cr compounds are presented in this section.

#### 5.1.1 Phase Stability: Formation Energies (Enthalpies of Formation) and Mixing Enthalpies

Regarding the CoCr‐based family of alloys, DFT calculations demonstrated that the cubic δ‐CoCr phase was energetically preferable over the *hcp* and *bcc* phases for Cr content equal to 62.5 at% and 75 at%, but the *hcp* structure was energetically preferable for 50 at% Cr.^[^ ^169^ ^]^ However, in this study, only non‐magnetic states were considered due to the assumption that the high Cr concentration leads to a vast reduction of the *T*_C_. The phase stability of σ‐CoCr from DFT calculations was investigated within the CALPHAD (CALculation of PHAse Diagrams) framework^[^ ^170^ ^]^ by explicitly considering spin polarization.^[^ ^171^ ^]^

As mentioned above, the FeCr system has historically attracted significant attention due to its relevance to stainless steels and remarkable mechanical properties. Such acquired knowledge is useful toward the overall understanding of various atomistic‐scale phenomena in the system related to other properties or research directions, such as the magnetic applications examined here. For instance, the phase diagram of the ferromagnetic Fe–Co–Cr alloy, along with its microstructural and micromagnetic modulations under thermal treatment and external magnetic field, were computationally investigated within the CALPHAD framework but based on experimental equilibrium phase diagram measurements.^[^ ^172^ ^]^ DFT calculations demonstrated that the anomalous stability of the ferromagnetic *bcc* FeCr with 6–9 Cr at% content and the negative sign of the mixing enthalpy occurred only for the FM phase and could be associated with variations of the electronic density of the majority spin channel at the Fermi level, which led to a tendency toward spinodal decomposition.^[^ ^173^ ^]^ The phase stability of σ‐FeCr against the α‐phase was also demonstrated by ab initio simulations, showing that the latter always precipitates from the liquidus due to the lower formation energy of the former.^[^ ^174^ ^]^ The interesting behavior of Cr mentioned in the previous sections has also motivated a thorough investigation of the energetics of Cr defects in *bcc* Fe and the corresponding impact to the atomic magnetic moments, revealing effects such as the repulsive character of Cr–Cr interactions for small Cr clusters of sizes up to four atoms.^[^ ^175^ ^]^

Moreover, ab initio simulations were used to investigate the short‐range ordering in Ni‐rich NiCr alloys, revealing the impact of small, strain‐induced interactions (as well as of extended multisite interactions) and the dependence of chemical interactions to the local environment, besides the dependence to concentration.^[^ ^176^ ^]^

Finally, the formation energies of nickel chromite (NiCr_2_O_4_) and of other related stoichiometric spinels (NiFe_2_O_4_, Fe_3_O_4_, FeCr_2_O_4_, CrFe_2_O_4_, ZnFe_2_O_4_, and ZnCr_2_O_4_), as well as their mixing enthalpies considering normal–normal, inverse–inverse, and normal‐inverse configurations, and the energetics of intrinsic anti‐site, Frenkel, and Schottky defects (as well as defects due to non‐stoichiometric interactions) were calculated in ref. [177].

#### 5.1.2 Atomic Magnetic Properties (Local Magnetic Moments, Exchange Coupling Constants, and Magnetocrystalline Anisotropy Energy)

The magnetic phase, local magnetic moments, exchange coupling constants, and electronic partial density‐of‐states (PDOS) of pure and bi‐doped CoCr_2_O_3_ were calculated in ref. [178], in agreement with experimental synthesis and characterization of Co_(1−_ *_x_*_)_Bi*_x_* Cr_2_O_4_ NPs (*x* = 0, 0.05, and 0.10). Further, the impact of charged Ni substitution at the +2 oxidation state (Ni_II_) in the α‐Cr_2_O_3_ (chromia) phase to the electronic DOS and atomic magnetic moments, for the determination of the properties of the ferromagnetic shell layer in experimentally fabricated α‐Cr_2_O_3_@α‐Ni_0.58_Cr_1.42_O_2.88_ inverted core–shell NPs was assessed in ref. [69]. Calculations demonstrated the Mott–Hubbard insulating behavior of the shell.^[^ ^69^ ^]^ In addition, the important role of assessing magnetic properties, regardless of application, was demonstrated in ref. [179] as significant magnetic fluctuations were observed in DFT calculations on paramagnetic austenitic steels (18 at% Cr, 8–12 at% Ni). These fluctuations were found to contribute to the stabilization of the austenitic phase against the *fcc*–*hcp* martensitic transformation close to the RT. Therefore, the strong, experimentally observed energy dependence of the stacking fault energies in these compounds could be attributed to the observed large disorders in local magnetic moments.^[^ ^179^ ^]^

The interplay between magnetic and various thermodynamic and mechanical properties is also relevant for Fe–Cr alloys; particularly because formations such as Cr nanoclusters in Fe bcc or Fe nanoclusters in bcc Cr are commonly observed during the α–α’ decomposition. Hence, the magnetic behavior of these nano‐inclusions was studied by DFT calculations, showing that in Fe‐embedded Cr clusters, collinear magnetization arose from the interfacial Fe–Cr coupling which dominated over AFM Cr–Cr interactions, while in Cr–embedded Fe clusters, noncollinear magnetic states were prevalent, at least in small clusters, as a means of relaxing the magnetic frustration.^[^ ^180^ ^]^ Additional Monte Carlo (MC) magnetic cluster expansion simulations extended these results to larger cluster sizes, revealing that non‐collinear magnetism can also be observed in larger Fe‐embedded Cr clusters; the latter were used for assessing the temperature dependence of these systems, potentially attributing the experimentally observed drop of magnetization at high temperatures to nanostructural changes.^[^ ^180^ ^]^

#### 5.1.3 Reaction / Adsorption Energetics

**Figure 12:**
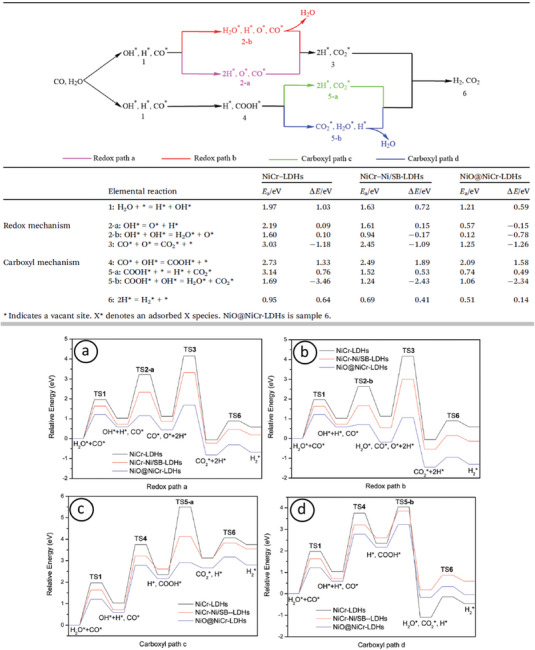
Top: reaction path on hybrid NiO@NiCr‐LDHs and tabulated corresponding activation energy and reaction energy change in each step. Bottom: reaction energy variation for the WGSR over various samples: redox mechanisms (a,b) and carboxyl mechanisms (c,d). Reproduced with permission.^[^ ^182^ ^]^ Copyright 2020, Royal Society of Chemistry.

Understanding the interaction of *M*–Cr alloys and Cr‐based spinel oxides with other molecules is important not only for assessing their interactions with ambient air and, thus, their chemical stability, but also for catalytic applications in which these materials have shown promising performance. In this direction, DFT simulations complemented by experimental evaluations were used to demonstrate the use of the CuCr_2_O_4_, NiCr_2_O_4_ spinel oxides as catalysts for NO removal by selective reduction with NH_3_.^[^ ^181^ ^]^ Activation energies and reaction energy changes for oxidation–reduction and carboxyl mechanisms for water shift gas reactions (WSGR) by using NiCr, NiCr–Ni, and NiO@NiCr layered double hydroxides (LDH) as catalysts were calculated in ref. [182] (**Figure** **12**). Moreover, ab initio MD simulations were used to propose an oxidation mechanism of CoCr (0001) surface with Cr content of 33 at% via the formation of an intermediate, cobalt‐rich amorphous matrix.^[^ ^183^ ^]^ Cr was consequently found to surface segregate and, in doing so, create vacancies which enabled oxygen to diffuse into the material.^[^ ^183^ ^]^

### 5.2 Molecular Dynamics / Monte Carlo

On the other hand, the classical approach of numerically integrating the equations of Newtonian dynamics, as employed in MD simulations, is an invaluable tool for exploring the properties of matter at greater size scales (compared with ab initio methods) due to its relatively simple theoretical background. To compensate for the lack of explicitly describing the electronic structure, the interactions between atoms for a given system are represented through an appropriately formulated and parametrized inter‐atomic potential (IAP). Determining the most suitable form of IAP for a specific configuration is one of the most critical and sensitive tasks in MD simulations. IAP parametrization is traditionally performed by fitting a property of interest to experimental or ab initio data. However, the first case is often problematic due to difficulties in experimentally determining the property of interest with the desired degree of accuracy (or even at all), whereas the second one is impacted by the limitation of ab initio simulations, such as the treatment of the strongly correlated 3*d* electrons in the case of the *M*–Cr system. An additional hurdle for describing materials such as *M*–Cr compounds arises due to their complex magnetic properties, which are also not explicitly considered in MD; their impact should be indirectly accounted for during the fitting of the IAP and this poses an extra challenge. However, MD simulations have overall been extensively used to explore properties and phenomena such as mechanical behavior, segregation, diffusion, and growth (as described, for instance, in refs. [41, 184, 185]) and they have given accurate results for a wide variety of compounds, including the *M*–Cr system.

#### 5.2.1 Interatomic Potentials

The parametrization of IAPs for the Fe–Ni–Cr system has been the subject of many studies as it is a main ingredient of steels with excellent stability and corrosion resistance; a particular application is for structural components of nuclear reactor vessels. As radiation‐induced degradation is a notorious challenge in such extreme environments, understanding of atomistic‐level effects such as defect formation, segregation, and diffusion is important for specifying the limitations of currently used compounds; as such, it has motivated a significant amount of work in the literature. A list of key advances in IAP development for each family of *M*–Cr materials is presented here in reverse chronological order.

##### FeNiCr

- Embedded atom method (EAM) potential parametrized by DFT calculations, focused on appropriately reproducing phase stability and thermodynamic properties at elevated temperatures (300 and 800 K), stacking fault energies, and lattice and elastic parameters.^[^ ^186^ ^]^
- EAM IAP parametrized by DFT calculations, with emphasis on accurate reproduction of short‐range interactions for simulating high‐energy cascades,^[^ ^187^ ^]^ by applying a short‐range refitting procedure described in ref. [188].
- EAM‐like IAP from DFT calculations for describing the *bcc* FeNiCr phase.^[^ ^189^ ^]^ The DFT calculations were focused on the energetics of small NiCr interstitials and small vacancy clusters in *bcc* Fe, and the potential was used in MC simulations for investigating the solubility of Ni and Cr in thermal equilibrium. Previous work on *fcc* FeNiCr was in ref. [190] (“EAM‐13”), where the focus was on reproducing vacancies, stacking faults, and point defects energies at the cost of less accurate elastic constants, and in ref. [191] (“EAM‐11”), which was claimed by the authors to be better suited for interactions between dislocations and defects but had several drawbacks such as inconsistencies in reproducing DFT point defect energetics. Moreover, Zhou et al.^[^ ^186^ ^]^ reported that “EAM‐13” incorrectly predicted Cr to be unstable in the austenitic phase and to separate at high temperatures (which contradicts known evidence that this phase should remain homogeneous and stable at high temperatures); while “EAM‐11” yielded a decreasing stacking fault energy with increasing Ni concentration, which contradicts both experimental and DFT findings.
- Second‐nearest‐neighbor modified EAM IAP for Fe–Ni, Ni–Cr, and Fe–Ni–Cr from experimental data of mixing/formation enthalpy, lattice parameters, and elastic constants.^[^ ^192^ ^]^ The potential was evaluated by comparing values of enthalpy of formation or mixing of the disordered phase and the enthalpy of mixing of the liquid phase from experimental and CALPHAD data and self‐ and vacancy diffusion from experimental data. The authors reported an improvement in performance compared with previous works, but still with some discrepancies, compared with reference experimental data.

##### FeCr

- Tersoff IAP with emphasis in accurately modeling the surface segregation of Cr in Fe.^[^ ^193^ ^]^
- Machine learning (ML) based IAP by harvesting the power of artificial neural networks (ANNs) via the Gaussian approximation potential (GAP) method.^[^ ^194^ ^]^ The IAP was fitted on DFT data, with emphasis on data produced for nudged‐elastic band (NEB) calculations for defect migration. The authors used this potential to perform atomistic kinetic‐MC thermal annealing simulations and found results in closer agreement to experimental findings compared with previous related EAM IAPs.
- Reactive force field (ReaxFF) IAP for the Fe–Cr–O–S system parametrized from DFT calculations.^[^ ^195^ ^]^
- Comparison of the predicted defect formation energies between four different “traditional” IAPs and DFT calculations revealed significant discrepancies on the order of magnitude of a few eV.^[^ ^196^ ^]^ Deviations were reported to be particularly large near the Cr‐rich limit as this case was not considered during IAP parametrization, due to the interest for the Fe‐rich limit for applications. The four tested potentials were:
  a) EAM Fe–Cr IAP for Fe–Cr within the two‐band model, parametrized from experimental data. The IAP was able to reproduce the α/α’ miscibility gap and phonon entropy data at 300 and 1600 K.^[^ ^197^ ^]^
  b) Concentration‐dependent potential trained in DFT calculations of random ferromagnetic *bcc* Fe–Cr alloys. The formation energy of vacancies was found to be linearly dependent on Cr percentage when the latter was greater than 6 at%.^[^ ^198^ ^]^
  c) Bonny et al.^[^ ^199^ ^]^ revised the two band model based on the model of Olsson et al.^[^ ^200^ ^]^
  d) The two band model for α‐prime FeCr was trained on DFT data and tested against DFT predicted energies of substitutional and interstitial defects. This model was consequently used in kinetic‐MC simulations to predict the microstructural evolution of random FeCr to the α‐prime phase due to thermal aging.^[^ ^189^ ^]^
- Tersoff IAP parametrization for reproducing the properties of Fe and Cr carbides.^[^ ^201^ ^]^

##### NiCr

- Angular‐dependent potential (ADP)‐type variation of the EAM IAP, fitted from experimental measurements and DFT measurements on vacancy formation and surface energies.^[^ ^202^ ^]^ Properties calculated with this IAP were demonstrated to be in agreement with experimental and DFT predicted values, both for the single Ni and Cr metals and the NiCr alloy (DFT data); the IAP was also able to reproduce experimental phase diagram data.

#### 5.2.2 Investigations of Nanoscale Phenomena and Property Prediction

Besides IAP development, MD/MC simulations are widely used to investigate various effects and properties at the nanoscale. For instance, the four IAPs tested by Klaver et al.^[^ ^196^ ^]^ were used in many works; the authors stated some for prediction of phase diagrams,^[^ ^203^ ^]^ phase separation,^[^ ^204^ ^]^ homogeneous^[^ ^205^ ^]^ and heterogeneous^[^ ^206^ ^]^ precipitation, short^[^ ^207^, ^208^ ^]^ and long^[^ ^209^ ^]^ range order, impact of displacement cascades to defect properties,^[^ ^210^ ^]^ diffusivity of self‐interstitial clusters,^[^ ^211^ ^]^ Cr‐self interstitial interactions,^[^ ^212^ ^]^ Cr‐dislocation interactions,^[^ ^213^ ^]^ Cr precipitate‐dislocation interactions,^[^ ^214^ ^]^ Cr segregation at grain boundaries,^[^ ^215^ ^]^ radiation‐induced segregation,^[^ ^216^ ^]^ point defect recombination and annealing,^[^ ^217^ ^]^ and stability of vacancy clusters versus Cr content.^[^ ^218^ ^]^ Moreover, a study of the wet oxidation of the *bcc* Fe–Cr surface (Cr content at ≈20 at%) was performed^[^ ^219^ ^]^ by demonstrating the dissociative adsorption of water molecules. The proposed mechanism was based on the strong attraction of O from Cr surface atoms and the consequent segregation of Cr and diffusion of the adsorbent fragments, which eventually led to the formation of a variety of Cr and Fe oxides, hydroxides, and hydrates. Bohra et al. performed MD simulations using the “EAM‐13” FeNiCr two‐band EAM IAP (Bonny et al.^[^ ^190^ ^]^) to complement and verify experimental findings by demonstrating the presence of residual Cr being trapped in the core of core–shell Ni@Cr NPs, when the Cr content was 10–15 at% (see above, Section 4.2.2).^[^ ^68^ ^]^ MD analysis also verified the experimentally observed surface segregation of Cr in NiCr NPs of 5 at% Cr content.^[^ ^115^ ^]^

Kuronen et al. assessed the formation of Cr precipitates in FeCr alloys for Cr content > 10 at%^[^ ^220^ ^]^ using MC simulations with the 2011 IAP by Bonny et al.^[^ ^199^ ^]^ By increasing the Cr content, they found Cr atoms that were not able to reach the surface layer but were being attached to pre‐existing Cr precipitates instead. However, their results from DFT simulations showed that Cr would preferably form Cr‐containing surfaces at this Cr content. MD simulations for investigating the dissolution of Cr precipitates with diameters of 0.3, 1, and 3 nm in Fe and Fe‐15 at% Cr lattices under thermal ageing was performed.^[^ ^221^ ^]^ The authors used the two‐band EAM IAP by Olsson et al.^[^ ^200^ ^]^ fitted to ab initio data of mixing enthalpy, in which Cr contents less than 10% were associated with negative mixing enthalpy values, finding that Cr precipitates dissociated via vacancy exchange at temperatures between 1500 and 2000 K, while they remained stable at both low and intermediate temperatures (≈1000 K). An MD study on the impact of uniaxial tensile strain on cylindrical [001] Fe and FeCr nanowires was reported in.^[^ ^222^ ^]^ The authors used the original two‐band EAM IAP of Olsson et al.^[^ ^200^ ^]^ and the Tersoff IAP of Henriksson et al.^[^ ^201^ ^]^ and found that their configurations were elastically softer compared to their respective bulk structures, with a higher yield strength for thin Fe nanowires and a weakening effect with increasing Cr content or diameter. Twinning was also found to be the main deformation mechanism.

### 5.3 Atomistic Spin Dynamics and Micromagnetic Simulations

As shown above, the ab initio family of methods can be used to predict the behavior of the electronic and, consequently, of the magnetic structure on the atomistic level, while MD simulations can assess phenomena at up to a few hundreds of nm (or even thousands, with the latest HPC developments) but by treating the impact of the electronic structure indirectly. Atomistic spin dynamics (ASD) methods come into play in order to cover this gap of predicting the magnetic structure of a given system at larger scales than ab initio methods, by explicitly considering the atomic magnetic moments. The backbone of ASD methods is the spin Hamiltonian, which typically includes energy terms for the interaction between spins, the interaction of spins with an external magnetic field (the Zeeman term), and the spin anisotropy (which describes the preference of spins to align toward specific directions and is strongly dependent on the crystal lattice). The parametrization of the spin Hamiltonian is typically performed based on experimental data or by assuming the relative magnitude between parameters (mostly between anisotropy and exchange interaction constants), but also from DFT calculations. The time evolution of the spin Hamiltonian can be estimated by the appropriate time integration of the Landau–Lifshitz–Gilbert (LLG) equation,^[^ ^223^ ^]^ while thermal effects can be included by adding a thermal term according to the Langevin dynamics model, which effectively represents a Gaussian white noise with a width which increases with increasing temperature.^[^ ^224^ ^]^ Moving up one order of magnitude, micromagnetic simulations are used to assess phenomena close to the µm scale by entering the domain continuum approximation in which the atomistic structure is considered small enough to be ignored but the length scale resolution is fine enough to investigate the behavior of effects such as domain walls or spin vortices. This section includes some examples of the application of this type of simulation for *M*–Cr alloys.

**Figure 13:**
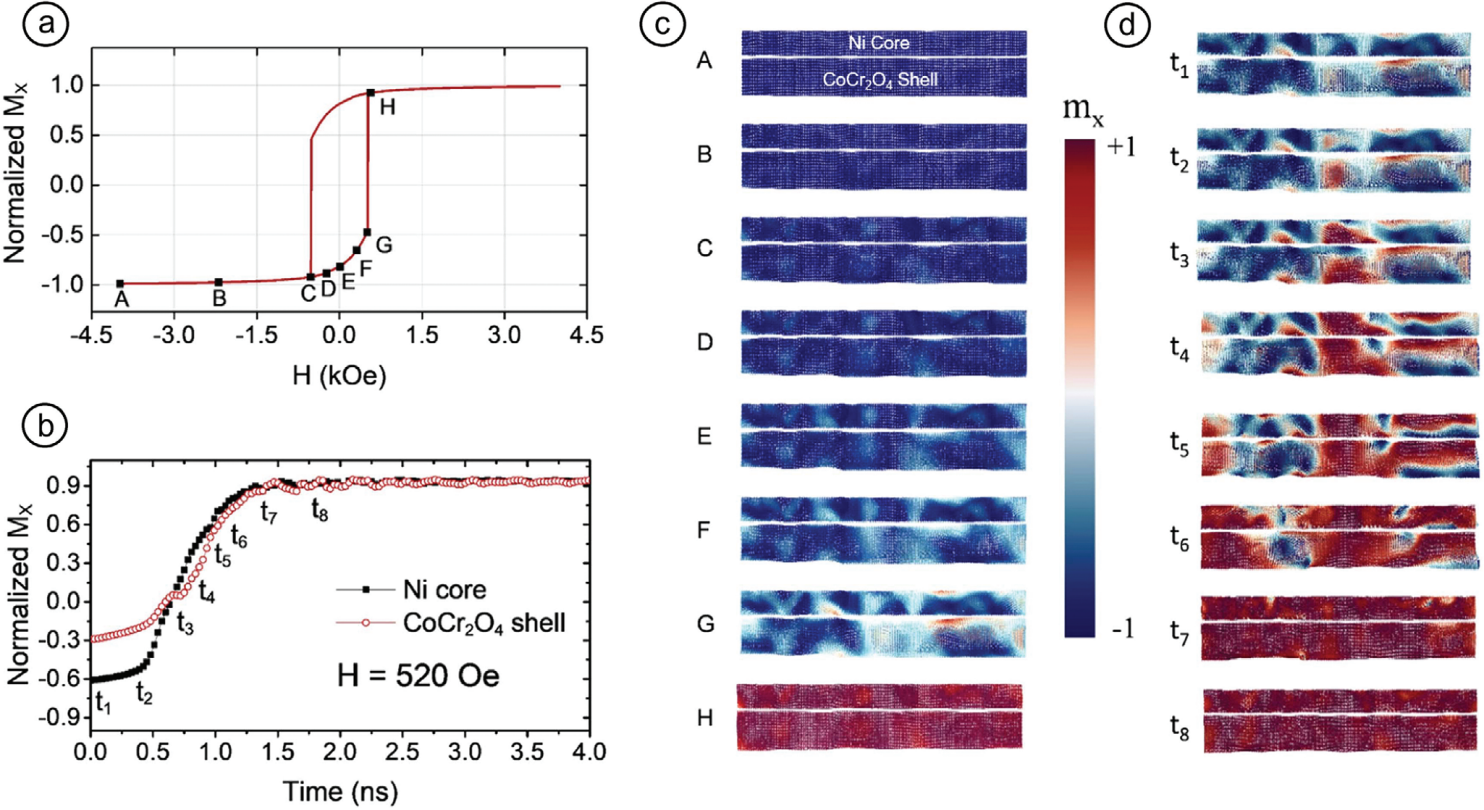
a) Hysteresis loop for an individual core–shell CoCr_2_O_4_/Ni NW at 0 K. b) Time‐dependent magnetization reversal process from point G to point H. c) A sequence of magnetization configurations captured at various magnetic fields during the magnetization reversal process. d) Transient magnetization configurations of the Ni core and CoCr_2_O_4_ shell at different time points from *t*_1_ to *t*_8_. Reproduced with permission.^[^ ^226^ ^]^ Copyright 2018, IOP Publishing.

Vojtech et al. performed micromagnetic simulations for complementing experimental results on FeCr alloys with 40 wt% Cr for estimating the magnetic response of the solid solution state compared with that of the phase‐decomposed state, by considering Cr‐rich precipitates in an Fe‐rich matrix and vice versa.^[^ ^225^ ^]^ In the first case, the magnetic response of the two states was similar and in good agreement with the experimental findings, while in the latter case, the authors reported a change in susceptibility between the two states, which was not verified by their experimental data.^[^ ^219^ ^]^ LLG‐based micromagnetic simulations on CoCr_2_O_4_@Ni core–shell nanowires (both on a single nanowire and a nanowire array) were performed^[^ ^226^ ^]^ for simulating the magnetization reversal (**Figure** **13**). The authors compared the hysteresis loops to those of a pure Ni nanowire and found that coercivity was not strongly dependent on temperature for their system.

**Figure 14:**
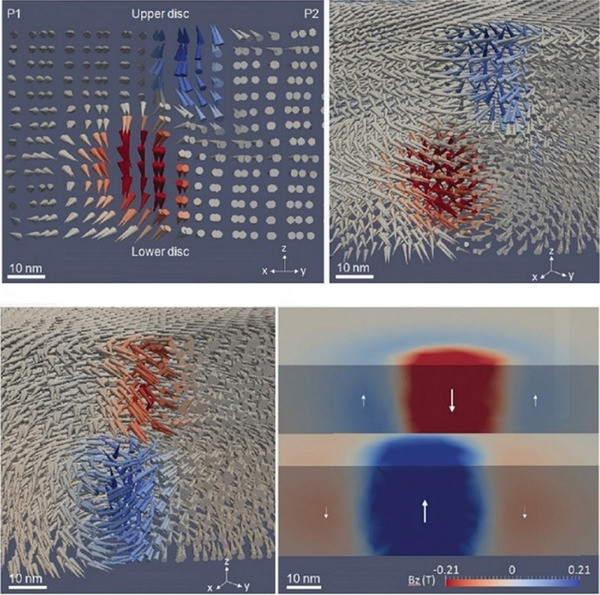
Top: 3D representation of reconstructed magnetic vortex cores, where the *z*‐directional components are denoted by blue (+*z*) or red (−*z*). (Left) Cross‐sectional magnetic vectors intersecting the P1−P2 line, with opposite *z*‐directions for upper and lower vortex cores. (Right) 3D view of the tail‐to‐tail vortex cores. Bottom: Micromagnetic simulation results illustrating counter‐clockwise configurations with stacked magnetic discs featuring opposite vortex cores. Initial magnetization was set to [$\bar{1}0\bar{1}$]. (Left) 3D view of vortex cores, showing *z*‐directional components in blue (+*z*) or red (−*z*). (Right) Mapping of *z*‐directional components around vortex cores indicated by blue (+*z*) or red (−*z*), surrounded by magnetic vectors with *z*‐directional components opposite to those of the vortex cores. Reproduced with permission.^[^ ^227^ ^]^ Copyright 2015, American Chemical Society.

**Figure 15:**
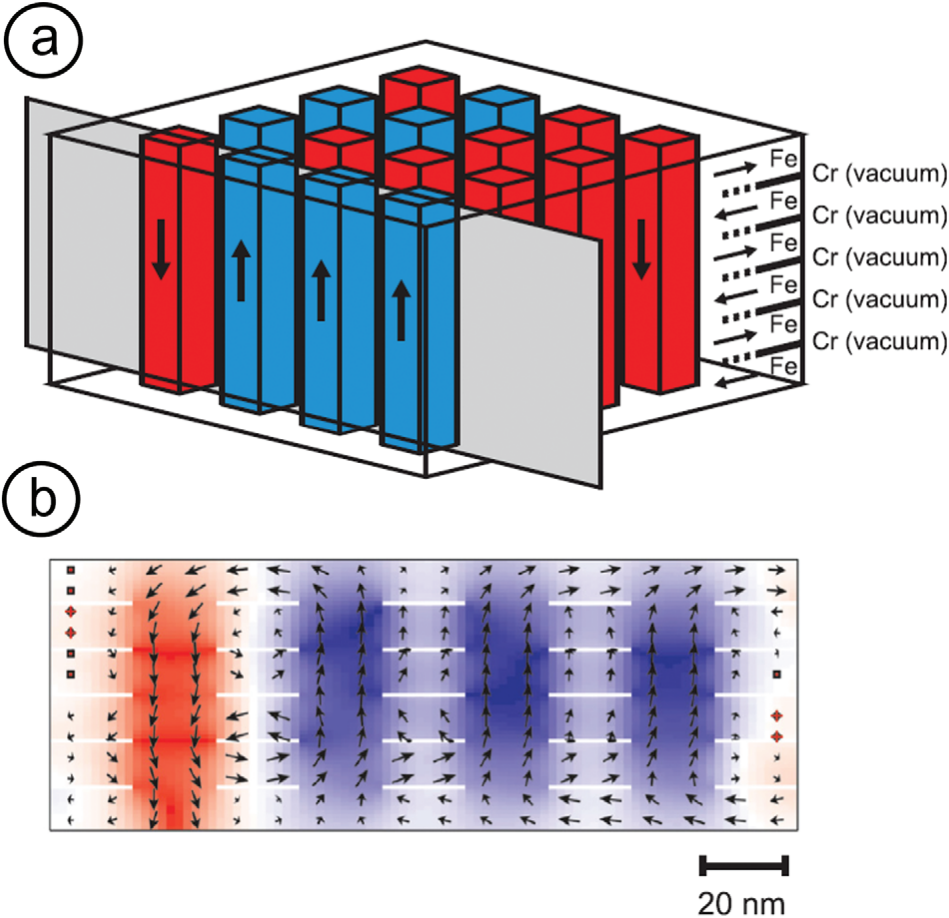
Micromagnetic simulation of a 4 × 4 array of 20 × 20 nm^2^ squares with a thickness of 65 nm, embedded into a Fe/vacuum multilayer structure consisting of six 10 nm thick Fe layers and five 1 nm thick vacuum layers representing the Cr. The AFM coupling is incorporated using bilinear and biquadratic coupling terms between neighboring Fe layers. a) Idealized image of the overall result. b) Slice of the actual numerical result (grey area marked in [a]). The perpendicular orientation of the magnetization of the elements is clearly observable, while the AFM coupling in the area between the elements remains predominantly intact (red color indicates magnetization pointing downwards; blue color indicates magnetization pointing upwards). Reproduced with permission.^[^ ^228^ ^]^ Copyright 2012, AIP Publishing.

LLG‐based micromagnetic simulations on Fe–Cr–Fe stacked FM discs on a nanoscale pillar were used to complement holographic vector field electron tomography measurements, a novel experimental imaging approach for 3D visualization of spin configurations (**Figure** **14**).^[^ ^227^ ^]^ Moreover, micromagnetic simulations of an AFM‐coupled Fe–Cr layered structure captured the magnetic structure of a proposed magnetic device.^[^ ^228^ ^]^ The results were used to show that the desired AFM behavior could potentially be achieved for even more densely packed structures than used in the experiments (**Figure** **15**).^[^ ^228^ ^]^ Finally, micromagnetic simulations were used^[^ ^229^ ^]^ to complement experimental analysis on carbon nanotube magnetic force microscopy probes, coated with CoFe and CoCr coatings, to investigate the impact of the coating material on the performance of the probe by assessing the saturation magnetization of each material.

On the other hand, Evans et al. performed ASD simulations for evaluating the impact of interfacial roughness on the exchange bias in AFM‐FM core–shell NPs.^[^ ^230^ ^]^ The exchange bias field was found to be heavily dependent on the local atomic magnetic moments at the interface but not on the relative magnitude of the AFM anisotropy (compared with the strength of the exchange interactions). The assessment of the SPM limit on core–shell NPs (of a different system, nevertheless) with FM core and AFM shell with ASD calculations was reported.^[^ ^231^ ^]^ The authors observed a pronounced energy barrier along the direction of the bias, in which the FM core was stable in the cases where the interfacial coupling was strong, and, therefore, suitable for thermal stability. Therefore, they proposed an approach for a magnetic recording device, which involved imposing a heat‐assisted bi‐stability in the system, allowing the switch of both the FM and AFM regions with a small external field upon heating to the Néel temperature.^[^ ^231^ ^]^ Finally, ASD was employed to study the transition between the AFM and the surface spin‐flop state in an epitaxial Fe/Cr (211) superlattice.^[^ ^232^ ^]^ The magnetization values produced by the model under varied external fields were in agreement with polarized neutron reflectivity experiments and revealed a first order transition toward a spin‐flop state in the surface for low angles between the easy axis and the external field.

From the above, it is evident that spin dynamics simulations are an invaluable tool that can be used to both assess nanoscale phenomena that cannot be investigated with experimental techniques and explore new geometries and configurations for the design and optimization of magnetic applications, as, for instance, shown in Figure 15. The length scales that can be approached with this family of methods are large enough to allow for the investigation of the most dominant magnetic effects; as seen in the previous paragraphs (for instance, in the novel imaging techniques used for Figure 14), despite the underlying theoretical simplicity, the results of this type of simulation can very accurately in reproducing experimental observations.

## 6 Conclusion

Our review article summarizes a multidisciplinary volume of key studies and contributes toward the realization of a broad spectrum of engineering and biomedical applications. As such, we aspire that it will be a significant addition to the literature for research communities in related fields.

### 6.1 Summary

The family of *M*–Cr NPs (where M is Fe, Co, Ni, or some combination of them) has attracted increased attention over the past years due to their exceptional and, most importantly, tunable magnetic properties. As such, they have been projected as candidate materials for high‐tech applications, including memory cells and recording heads, induced hyperthermia and drug delivery, and magnetic cooling.

In this review, we present the current state‐of‐the‐art and exciting prospects of *M*–Cr NP synthesis technologies, with a view to providing a comprehensive guide for future fundamental studies and next‐generation applications and devices. We distinguish where the use of *M*–Cr NPs can be particularly advantageous and where it is problematic. We also emphasize real‐world challenges and economical/environmental considerations. For example, solid‐state magnetocaloric materials can significantly reduce greenhouse gas emissions; further, dematerialization by using smaller quantities of non‐critical materials such as transition‐metal NPs may reduce supply risks or cost due to current global geopolitical issues.

The experimental fabrication of monodispersed NPs and the control of their structural characteristics are key factors in tuning their magnetic properties such as the Curie temperature or exchange bias. Composition (Cr content, in particular) is explored as a means to tailor the magnetic properties. However, a wide variety of nanoscale phenomena is also reported in the literature (formation of different crystalline phases, surface oxidation, segregation of constituent elements, coalescence, etc.) that can have a distinct impact on the macroscopic properties of the system. The different experimental approaches for the analysis and the tuning of the interplay between the aforementioned effects toward the desired properties for specific applications are discussed.

The role of computational methods is also of particular importance for understanding the underlying mechanisms that dominate the behavior of *M*–Cr NPs in different size scales. Ab initio simulations have provided insight regarding the magnetic properties of the fundamental constituent metals and oxides, predicting atomic magnetic moments, the magneto–crystalline anisotropy, and the exchange interaction coupling energy, as well as surface oxidation and the impact of strain. MD‐based methods have also been used extensively for reproducing and identifying phenomena related to the growth of NPs and also their shape, coalescence, and elemental segregation. MC and spin dynamics simulations offer another invaluable tool to the research of magnetic nanoclusters as they have been used for mapping atomistic properties onto macroscopic properties such as hysteresis and the *T*_C_.

### 6.2 Scope for Future Work

Although the present report reviews structural and magnetic features of *M*–Cr nanoclusters with a focus on magnetic cooling, spintronic, and biomedical applications, several additional aspects that could not be taken up here are also worth further investigation in future work:

- With the swift advancement of modern communication devices (5G–7G technologies and Internet of Things [IOT]), electromagnetic interference pollution has become a serious issue. The excess electromagnetic energy may have a negative impact on electronic instruments, the environment, or, most importantly, on human health. Therefore, microwave absorption materials, such as *M*–Cr based core‐shell nanoclusters, can provide a new avenue to solve this problem.
- For new directions in nano‐spintronics for sustainable and unconventional computing, magnetic elements such as *M*–Cr nanoclusters may be advantageous, especially in terms of data transport, for example, by propagation of domain walls or skyrmions in neuromorphic computing.
- Incorporation of these magnetic *M*–Cr nanoclusters into polymers, ceramics, or other materials can lead to the development of smart materials with programmable properties, such as magneto‐responsive materials for futuristic actuation and sensing applications.
- The investigation of electrochemical behavior such as high specific capacitance and charge/discharge rates of *M*–Cr core‐shell nanoclusters can render them promising contenders for electrode materials in supercapacitors, combining high levels of power delivery with high‐energy storage densities.
- From multicomponent HEAs (AlCoCrFeNi) to ternary alloys (CrFeNi) and binary (*M*–Cr) alloys, *M*–Cr is one of the fundamental compounds. Therefore, studying the thermal stability of these layers in various harsh atmospheres (high temperature, high pressure, and oxidation/reduction environment post annealing) provides insights into the altered structural, magnetic, and mechanical properties of the resulting nanocluster thin films for nuclear reactor wall coating.

## Conflict of Interest

The authors declare no conflict of interest.
